# On the Unreliability of Test–Retest Reliability

**DOI:** 10.1177/01466216251401213

**Published:** 2025-11-26

**Authors:** Domenic Groh

**Affiliations:** 1 Institute of Psychology, 9376 Otto von Guericke University Magdeburg, Magdeburg, Germany

**Keywords:** test-retest reliability, reliability coefficients, classical test theory (CTT), Monte Carlo simulation, psychometric bias

## Abstract

The Test–Retest Coefficient (TRC) is a central metric of reliability in Classical Test Theory and modern psychological assessments. Originally developed by early 20th-century psychometricians, it relies on the assumptions of fixed (i.e., perfectly stable) true scores and independent error scores. However, these assumptions are rarely, if ever, tested, despite the fact that their violation can introduce significant biases. This article explores the foundations of these assumptions and examines the performance of the TRC under varying conditions, including different sample sizes, true score stability, and error score dependence. Using simulated data, results show that decreasing true score stability biases TRC estimates, leading to underestimations of reliability. Additionally, error score dependence can inflate TRC values, making unreliable measures appear reliable. More fundamentally, when these assumptions are violated, the TRC becomes underidentified, meaning that multiple, substantively different data-generating processes can yield the same coefficient, thus undermining its interpretability. These findings call into question the TRC’s suitability for applied settings, especially when traits fluctuate over time or measurement conditions are uncontrolled. Alternative approaches are briefly discussed.

## Introduction

The reliability of measurement tools is a foundational concern in psychological research. Inferences about traits, behaviors, and processes depend on the extent to which instruments consistently capture the constructs they are intended to measure. Within psychology, the subfield of psychometrics focuses on assessing the validity and reliability of instruments, often using the framework of classical test theory (CTT). CTT has guided the development of reliability coefficients such as Cronbach’s alpha, split-half reliability, and the Test–Retest Coefficient (TRC). While the structural underpinnings of coefficients like Cronbach’s alpha and the split-half method have been extensively examined and critiqued (Sijtsma, 2008; Webb et al., 2006), the TRC has been predominantly examined on experimental grounds, which leaves its structural robustness underexplored. Despite these gaps, the TRC remains a foundational measure of reliability in behavioral science (Berchtold, 2016; Polit, 2014).

The TRC is based on the assumptions of perfectly stable true scores and the absence of systematic errors, both of which are conditions that are seldom met in real-world applications. Surprisingly, no research has investigated how the TRC performs when these assumptions are violated, which raises important questions about its validity and utility. This study seeks to address these issues by delineating the TRC’s theoretical foundations within the CTT framework and evaluating its reliability and feasibility through simulation.

The paper is organized as follows. First, the logic of the TRC is outlined within the framework of CTT, with a brief emphasis on its assumptions and derivations. Next, a simulation study examines the TRC’s performance under varying conditions, including true score stability and error score dependence. Finally, the implications of these findings are discussed, along with recommendations for the use and interpretation of the TRC in psychometric research.

## Reliability in Classical Test Theory

The framework of CTT posits that every observed score x on a measurement can be linearly decomposed into a true score τ and an error score ε :

(1) x=τ+ε

The true score τ is defined as the expected value E(x) of an individual’s observed scores over repeated, independent measurements. It therefore represents the hypothetical score an individual would receive if they were tested an infinite number of times under identical conditions. Any deviations from this fixed value are captured by the error term ε and thus reflect, per definition, unsystematic influences on the measurement. This implies that, for any given individual on any given measurement, the true score remains constant across administrations, and therefore the variance of their true score is zero (Lord & Novick, 2008).

Furthermore, CTT defines the expected value of the error scores E(ε) to be zero and asserts that the error scores are linearly independent with the true scores and themselves, i. e. zero covariance (Lord & Novick, 2008):

(2) $$Cov\left(\tau ,\varepsilon \right)=0\;and\;Cov\left(\varepsilon_{i},\varepsilon_{j}\right)=0\;\left(i\neq j\right)$$

Within CTT reliability is defined as the proportion of variance that is caused by the true score, divided by the total observed variance of the measurement (Lord & Novick, 2008):

(3) $${r_{\tau x}}^{2}={\left(\frac{Cov\left(\tau ,\tau +\varepsilon \right)}{\sqrt{Var\left(\tau \right)Var\left(x\right)}}\right)}^{2}={\left(\frac{Var\left(\tau \right)}{\sqrt{Var\left(\tau \right)Var\left(x\right)}}\right)}^{2}=\frac{Var\left(\tau \right)}{Var\left(x\right)}=\frac{{\sigma_{\tau }}^{2}}{{\sigma_{x}}^{2}}$$

The square root of this expression $r_{\tau x}$ is referred to as the reliability index. While elegant in formulation, this coefficient is not directly observable because true scores themselves are not observable. To address this limitation, several operational measures of reliability have been devised under the CTT framework, chief among them the TRC. The TRC is obtained by administering the same test twice to a sample, thereby yielding two sets of observed scores, $x_{1}$ ​ and $x_{2}$ , which correspond to the first and second administrations, respectively (Webb et al., 2006).

Because of the earlier assumptions about true and error scores, the TRC simplifies as follows:

(4) $$r_{x_{1}x_{2}}=\frac{Cov\left(x_{1},x_{2}\right)}{\sqrt{Var\left(x_{1}\right)Var\left(x_{2}\right)}}=\frac{Cov\left(\tau_{1},\tau_{2}\right)}{\sqrt{Var\left(\tau_{1}+\varepsilon_{1}\right)Var\left(\tau_{2}+\varepsilon_{2}\right)}}=\frac{Var\left(\tau \right)}{\sqrt{Var\left(x\right)Var\left(x\right)}}$$

where the simplification assumes that the true scores are invariant $\left(\tau_{1}=\tau_{2}=\tau \right)$ and that the error terms are uncorrelated with the true scores and with each other (i.e., Cov(τ,ε)=0 and $Cov\left(\varepsilon_{i},\varepsilon_{j}\right)=0$ ; Guttman, 1945).

Unlike single-administration coefficients, such as McDonald’s ω , Cronbach’s α , or the greatest lower bound (GLB), this has the benefit of being an exact measurement and not just a lower bound of reliability as long as the assumptions are met (Guttman, 1945; Zinbarg et al., 2005). The magnitude of the TRC is commonly interpreted such that scores around .7 are the lowest levels of *“acceptable”* reliability, scores above .8 are *“good”* reliability and scores above .9 are *“excellent”* reliability (Matheson, 2019).

## Factors Affecting the Test–Retest Correlation

While the preceding section showed that the TRC can, under ideal conditions, serve as an exact measure of reliability, this equivalence depends on a range of assumptions that are rarely fully, if ever, satisfied in practice. These assumptions stem not only from the theoretical framework of CTT itself but also from the statistical properties of the correlation coefficient—typically Pearson’s r —that is used to estimate the TRC. If these underlying assumptions are violated, the TRC may either underestimate or overestimate the true reliability of a measurement instrument. However, before the TRC’s performance under varying conditions can be evaluated, the key quantities influencing its estimation must first be specified.

The first and perhaps most straightforward condition is the requirement of an adequate sample size. As stated above, in CTT, the reliability of a measurement is quantified using the correlation coefficient. The standard error of the Pearson correlation coefficient is a function of both sample size and the magnitude of the correlation itself, meaning that smaller true correlations require even larger samples to achieve stable estimates (Bowley, 1928). When sample sizes are too small for a given correlation magnitude, the sample correlation coefficient becomes an unstable estimate of the true population value. As a result, even a measurement with high true reliability may appear less (or more) reliable due to sampling variability. Notably, simulation studies indicate that, for correlations as large as r=.7 , a sample size of approximately 130 experimental units is required for the 95% confidence interval of the correlation estimate to stabilize at a width of w=.10 (Schönbrodt & Perugini, 2013). Lower correlations, and thus also lower reliability levels, necessitate even larger samples to achieve comparable estimation precision.

A second critical assumption of the TRC concerns the temporal stability of the trait being measured. As outlined in the preceding section, CTT defines the true score as the expected value of observed scores across repeated measurements. By this definition, the true score is assumed to be constant for each individual, and any deviations between test administrations are attributed solely to random error. However, psychological traits often exhibit some degree of intra-individual variation over time, even when labeled as “stable.” A longstanding distinction exists between psychological traits, which are considered relatively enduring dispositions (e.g., cognitive ability and personality), and psychological states, which reflect more transient experiences (Fridhandler, 1986). By their very nature, states fluctuate across time and thus violate the assumption of fixed true scores. Even among stable traits, empirical retest correlations over time commonly fall within a range of r ≈ .6 to .9 (Asendorpf & Wilpers, 1998; Breit et al., 2024; Costa et al., 2012; Rantanen et al., 2007; Scharfen et al., 2018). These estimates are not derived from a single method but span a variety of analytical techniques, including simple test–retest correlations, average inter-measure correlations, latent growth models, and meta-regressions. Regardless of method, they suggest that, while stable traits may approximate temporal invariance, they do not fulfill the strict CTT assumption of unchanging true scores.

A third factor influencing the TRC is the relative contribution of true and error score variances to the total observed variance. As mentioned above, in psychological testing, scores from repeated administrations typically contain both components. Again, under the assumptions of CTT, the true score is defined as fixed for each individual, making any observed fluctuations attributable solely to error. When this condition holds, changes in the TRC reflect differences in error variance: higher error variance reduces the TRC, while lower error variance increases it. This follows from the structure of the correlation formula, where error variance inflates the denominator but does not affect the covariance in the numerator. If, however, true scores also fluctuate over time, they contribute additional variance to the denominator, thereby reducing the TRC even further. The TRC would then become a joint function of both temporal stability and the relative proportions of true and error score variance. This can also be demonstrated formally. Given two observed scores from repeated administrations,

(5) $$x_{1}=\tau_{1}+\varepsilon_{1}\;\text{and}\;x_{2}=\tau_{2}+\varepsilon_{2}$$

and assuming that the true and error components behave as stated in equation (2) the test–retest correlation is:

(6) $$r_{x_{1}x_{2}}=\frac{Cov\left(x_{1},x_{2}\right)}{\sqrt{Var\left(x_{1}\right)Var\left(x_{2}\right)}}$$

Using the decomposition (5) and the independence assumptions, the covariance reduces to:

(7) $$Cov\left(x_{1},x_{2}\right)=Cov\left(\tau_{1}+\varepsilon_{1},\tau_{2}+\varepsilon_{2}\right)=Cov\left(\tau_{1},\tau_{2}\right)$$

Assuming equal variance for all components $Var\left(\tau_{1}\right)=Var\left(\tau_{2}\right)=\sigma_{\tau }^{2}$ and $Var\left(\varepsilon_{1}\right)=Var\left(\varepsilon_{2}\right)=\sigma_{\varepsilon }^{2}$ , to reflect that both observed scores are taken from the same population with the same measurement, it follows that:

(8) $$Var\left(x_{1}\right)=Var\left(x_{2}\right)=\sigma_{\tau }^{2}+\sigma_{\varepsilon }^{2}$$

$$Let\;r_{\tau_{1}\tau_{2}}=Corr\left(\tau_{1},\tau_{2}\right).\;Then$$

(9) $$Cov\left(\tau_{1},\tau_{2}\right)=r_{\tau_{1}\tau_{2}}\sigma_{\tau }^{2}$$

Substituting equations (7) to (9) into equation (6) then yields:

(10) $$r_{x_{1}x_{2}}=r_{\tau_{1}\tau_{2}}\times \frac{\sigma_{\tau }^{2}}{\sigma_{\tau }^{2}+\sigma_{\varepsilon }^{2}}=r_{\tau_{1}\tau_{2}}\times {r_{\tau x}}^{2}$$

thus demonstrating that, when trait stability is imperfect, the TRC is no longer equal to reliability (cf. equation (3)) but is instead the product of trait stability and the variance ratio. In other words, both instability and error variance attenuate the TRC simultaneously.

Beyond their proportional relationship, the absolute size of these variance components also shapes the distribution of observed scores. As total variance increases, the spread of scores becomes wider, making extreme values more likely. This is particularly problematic in small samples, where outliers can disproportionately influence statistical estimates and reduce the stability of the TRC. Broader distributions often exhibit heavier tails, meaning that even a small number of extreme observations can distort reliability estimates. Large sample sizes may still be required to achieve stable results, even when working with relatively light-tailed distributions, especially when population parameters must be estimated from empirical data (Wilcox, 2010). In such contexts, the TRC becomes more volatile, less reflective of true reliability, and more sensitive to random fluctuations.

Lastly, the TRC also depends on the independence of error scores. As discussed above, CTT assumes that error scores are random and independent across measurements. When error scores remain independent, they do not contribute to the correlation between repeated measurements. However, if error scores become dependent, meaning factors from the first measurement influence the second measurement, this dependency introduces covariance between the scores, which increases the resulting TRC. These errors are known as systematic errors because they arise from consistent factors related to the testing situation, sample characteristics, training effects, or other shared influences between the measurements (Polit, 2014). Unlike random error, which only reduces the reliability estimate, systematic error inflates the TRC by confounding the true score signal with extraneous variance that is shared across administrations. This makes it increasingly difficult to disentangle genuine reliability from the consistency of shared influences and makes a clear interpretation of the TRC impossible.

This too can be formally demonstrated. Starting from the classical decomposition of observed scores (equation (5)), the assumption that error terms are uncorrelated across time is now relaxed. The covariance between observed scores then becomes:

(11) $$Cov\left(x_{1},x_{2}\right)=Cov\left(\tau_{1},\tau_{2}\right)+Cov\left(\varepsilon_{1},\varepsilon_{2}\right)$$

Assuming, as before, equal variances for all components it again follows that:

(12) $$Var\left(x_{1}\right)=Var\left(x_{2}\right)=\sigma_{\tau }^{2}+\sigma_{\varepsilon }^{2}$$

Using the same logic as in equation (9), the true score and error covariances can be expressed as:

(13) $$Cov\left(\tau_{1},\tau_{2}\right)=r_{\tau_{1},\tau_{2}}\times \sigma_{\tau }^{2},\;Cov\left(\varepsilon_{1},\varepsilon_{2}\right)=r_{\varepsilon_{1},\varepsilon_{2}}\times \sigma_{\varepsilon }^{2}$$

Substituting equations (11) to (13) into the formula for the TRC yields:

(14) $$r_{x_{1}x_{2}}=\frac{r_{\tau_{1},\tau_{2}}\times \sigma_{\tau }^{2}+r_{\varepsilon_{1},\varepsilon_{2}}\times \sigma_{\varepsilon }^{2}}{\sigma_{\tau }^{2}+\sigma_{\varepsilon }^{2}}=r_{\tau_{1}\tau_{2}}\times \frac{\sigma_{\tau }^{2}}{\sigma_{\tau }^{2}+\sigma_{\varepsilon }^{2}}+r_{\varepsilon_{1},\varepsilon_{2}}\times \frac{\sigma_{\varepsilon }^{2}}{\sigma_{\tau }^{2}+\sigma_{\varepsilon }^{2}}$$

Thus, demonstrating, that when error dependence is introduced, the TRC becomes the weighted sum of true score stability and error score dependence, with each component scaled by its relative contribution to total variance. As a consequence, the TRC no longer reflects a pure estimate of reliability, but rather a combination of genuine signal and shared systematic error.

Taken together, the TRC is influenced by several factors: sample size, the stability of the measured trait, the variance of the true score, the variance of the error score, and the independence or dependence of errors. In any given study, these assumptions may be violated to varying degrees and may even interact with one another. While numerous experimental investigations of the TRC exist (e.g., Polit, 2014), no study to date has systematically examined how the TRC performs when these factors are manipulated. Therefore, this study simulates data under varying conditions of sample size, true score stability, and the variances of both true and error scores to explore the TRC’s performance under realistic conditions.

## Simulation Studies

To systematically investigate the conditions under which the TRC provides stable and accurate estimates of reliability, two separate simulation studies were conducted. Study 1 examined the effects of sample size, variance ratios, and true score stability under the assumption of independent error scores. Study 2 extended this by introducing correlation between error scores to assess how dependent error structures affect the TRC. Simulations were carried out using R (R Core Team, 2024).

For both studies, 1,000 samples were simulated per condition combination. The average correlation across these 1,000 datasets served as an estimate of the TRC’s accuracy, while the standard deviation of the correlations was used as a measurement of coefficient stability. This setup rests on a key statistical property of the correlation coefficient. Pearson’s r can be expressed as the mean of the cross-products of the z-scores of two variables (Rodgers & Nicewander, 1988), which places it under the Central Limit Theorem. As such, with sufficient sample size, Pearson’s r tends to follow an approximately normal distribution (Goldstein, 2010).

This property allows the standard deviation of r to serve as a meaningful indicator of stability. In a normal distribution, roughly 68% of values lie within one standard deviation of the mean, and about 95% within two. Following Schönbrodt and Perugini’s (2013) usage of confidence intervals, this study defines stability based on how tightly the distribution of TRC values clusters around a given reliability threshold. If the TRC is centrally located within one of the conventional reliability categories (i.e., *“acceptable”* ≥ .70, *“good”* ≥ .80, and *“excellent”* ≥ .90; Matheson, 2019), its distribution should not extend significantly beyond that range’s boundaries. A coefficient is considered to have *“good”* stability if 68% of the distribution remains within the same reliability range, and *“excellent”* stability if 95% does. Accordingly, a standard deviation of .05 or less is considered *“good*,” while .025 or less is considered *“excellent”*.

Following Lord and Novick (2008), reliability is conventionally quantified using Pearson’s *r*, which is also the standard metric employed in most test–retest manuals (Polit, 2014). Accordingly, Pearson’s *r* was chosen to estimate the TRC in the simulations. To provide a baseline “best-case scenario,” normally distributed variables were generated, as many of the classical distributional results for *r* (e.g., its sampling variance and the Fisher *z* transform) are derived under this assumption. Departures from normality are known to bias estimates of *r* (Bishara & Hittner, 2014; Kowalski, 1972), so the normal model offers a clear benchmark for evaluating performance while reflecting the approach most commonly used in practice.

Both the code and the graphics used to justify the assertion of normality for both studies are available at https://osf.io/x7ptw/.

### Study 1: Method

The first study examined how the TRC performs under varying conditions of sample size, variance ratio, and true score stability. Both the true score (*τ*) and the error score (*ε*) were generated from normal distributions across nine variance groups, with variances ranging from 1 to 9. The mean of all error score distributions was set to 0, while the mean of the true score distributions was arbitrarily set to 10 to distinguish them meaningfully from the error terms.

To simulate changes in true score stability, the correlation between the two true score variables (*τ*_1_ and *τ*_2_) was varied from 1.0 to .5 in increments of .1. As discussed, this reflects the typical range of stability observed in psychological traits. All *τ*- and *ε*- variables were simulated using the *rnorm_multi* function from the R package *faux* (DeBruine, 2023). In the fixed true score condition (i.e., perfect stability), a single *τ*_1_ variable was generated using *rnorm* and copied to serve as *τ*_2_. Observed scores (*x*_1_ and *x*_2_) were computed by summing the respective true and error scores (*x* = *τ* + *ε*). These observed variables were then correlated to estimate the TRC. This procedure was repeated for sample sizes of 2 to 1000. For each condition, 1,000 replications were run, and results are reported as the mean and standard deviation across replications.

The variables manipulated in Study 1 were as follows:

 (1) Sample size: 2 to 1000
 (2) True score variance: 1, 2, 3, 4, 5, 6, 7, 8, and 9
 (3) Error score variance: 1, 2, 3, 4, 5, 6, 7, 8, and 9
 (4) True score stability: Correlation between *τ*_1_ and *τ*_2_ from .5 to 1.0 in steps of .1

### Study 1: Results

**Figure 1.**
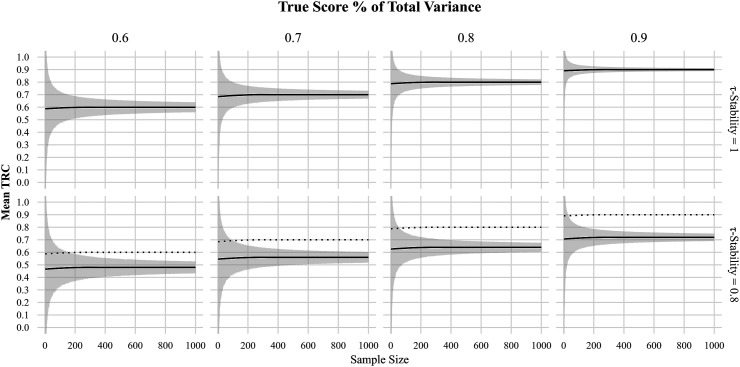
Simulation study results showing mean estimated correlations (mean TRC) over sample sizes (0 1000) for four variance ratios (0.6–0.9) at two true score stability levels (τ = 1 vs. τ = 0.8). Gray shaded ribbons represent 95% confidence intervals around each LOESS fit. The dotted line in the lower (τ = 0.8) panels overlays the τ = 1 trajectory for direct comparison.

Figure 1 displays how the mean TRC develops as a function of sample size and variance ratios across two τ -stability levels. Each panel presents simulation results for a specific variance ratio (ranging from *“unacceptable”* to *“excellent”* reliability). The top row shows that when true scores are perfectly stable, the TRC becomes a perfect estimator of reliability. The shaded area, representing the 95% confidence interval, also indicates that higher levels of reliability reach *“good”* ( $SD_{TRC}$ < .05) or even *“excellent”* ( $SD_{TRC}$ < .025) coefficient stability much faster than lower reliability levels.

These threshold criteria are formalized in Table 1, which provides the minimum sample sizes required to achieve good or excellent TRC coefficient stability across all combinations of variance ratio and τ -stability. For instance, a reliability of .9 (variance ratio 9:1) with perfect trait stability requires fewer than 30 participants to reach good stability.

**Table 1.** Recommended Minimum Sample Sizes for Different Variance Ratios Across True Score Stability Levels for Good or Excellent Stability

	τ -Stability											
	1				0.9				0.8			
Ratio	0.6	0.7	0.8	0.9	0.6	0.7	0.8	0.9	0.6	0.7	0.8	0.9
Criterion												
Excellent	855	617	382	199	773	536	349	188	847	676	491	351
Good	158	106	52	20	200	145	91	53	228	175	138	93

	0.7				0.6				0.5			
Ratio	0.6	0.7	0.8	0.9	0.6	0.7	0.8	0.9	0.6	0.7	0.8	0.9
Criterion												
Excellent	992	842	714	541	+999	+999	899	743	+999	+999	+999	953
Good	256	219	177	139	298	262	225	190	312	288	266	243

*Note. Criterion represents the SD threshold: Excellent = SD < .025 and Good = SD < .05.*

Ratio represents the true score variance divided by total variance.

In contrast, when true score stability (further also referred to as τ-stability) drops to .7, more than 500 participants are required, even at the same measurement reliability. Similarly, when τ -stability drops to .5 even excellent reliability demands samples larger than 500 for excellent coefficient stability.

The bottom row of Figure 1 illustrates what happens when τ -stability decreases to r = .8. Even under *“excellent”* reliability conditions (e.g., a 9:1 variance ratio), the TRC becomes systematically biased, thus underestimating reliability by approximately .18. This bias is strongest at high reliability and becomes less pronounced as the reliability decreases. For example, in Figure 1, the bias at a reliability of 0.6 (variance ratio 6:4) is only .12. The widening confidence intervals further show that both lower τ -stability and lower reliability increase the required sample size for stable TRC estimation.

**Figure 2.**
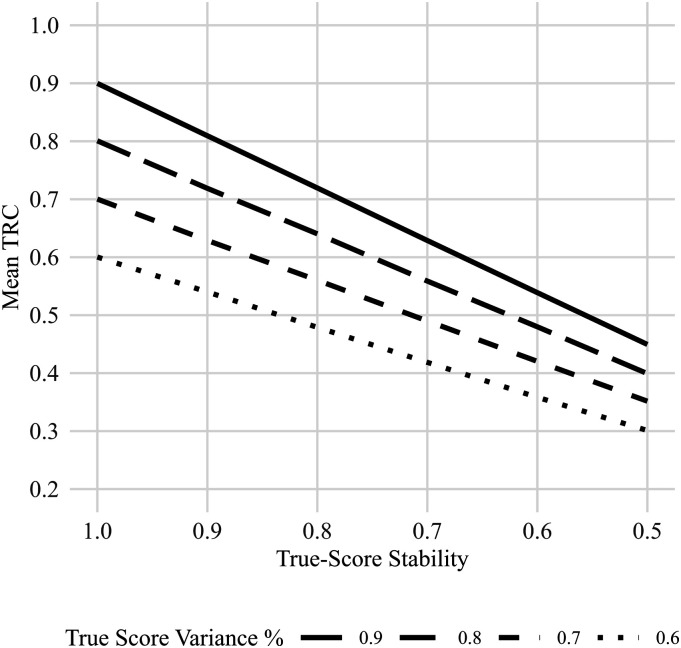
Relationship between mean estimated correlation (mean TRC) and true score stability at a fixed sample size of 1000, across four reliability levels.

The exact amount of bias, that is, the amount with which the TRC deviates from its underlying reliability, is quantified in Table 2. Figure 2 further visualizes the above-mentioned bias by plotting the mean TRC estimates at n=1000 across varying levels of true scores stability for four different true-to-error variance ratios. The results reveal a linear decline in the TRC as the stability of τ -stability decreases, which is consistent with the theoretical prediction that, under non-ideal conditions, the TRC approximates the product of trait stability and reliability. As τ -stability declines, the lines also begin to converge, thus indicating that the TRC becomes less sensitive to differences in reliability when traits are unstable. Exact values for selected combinations of τ -stability and reliability are provided in the supplementary material (Supplemental Table 1 and 2). Notably, even a measurement with excellent reliability (e.g., .9) can fall below the nominal acceptability threshold of .7 when trait stability declines to between .8 and .7, despite being factually highly reliable.

**Table 2.** Deviation of TRC Estimates from Actual Reliability Across Stability Condition at *n* = 1000

Stability	1	0.9	0.8	0.7	0.6	0.5
*Ratio*						
0.9	0	.09	.18	.27	.36	.45
0.8	0	.08	.16	.24	.32	.40
0.7	0	.07	.14	.21	.28	.35
0.6	0	.06	.12	.18	.24	.30

Lastly, contrary to the assumption stated in section 3, the simulations reveal that the absolute size of the true and error score variances does not influence TRC behavior beyond their relative ratio. That is, neither the degree of bias nor the variability in TRC estimates is affected by how large or small the variances are in absolute terms (cf. Supplemental Table 3). Instead, the TRC’s behavior is fully governed by the variance ratio and trait stability.

### Study 2: Method

The simulation for Study 2 mirrored that of Study 1, with the main difference being that error scores were drawn from correlated distributions rather than independent and identical ones, as detailed in Study 1.

As in Study 1, observed scores were calculated by summing the respective true and error scores (cf. Method Study 1). This was varied across three levels of error score correlation (.1, .3, and .5) and five levels of τ -stability (1, .9, .8, .7, and .6). The selected stability values were informed by the results of Study 1, where noticeable bias in TRC estimates began to emerge around a τ-stability of .8. While a τ -stability of .9 may be considered borderline acceptable, .8 appeared to mark a turning point at which the estimates became increasingly biased. The levels of error score dependence were intended to represent a small level of systematic error. To that end, an error score dependence of .1 represents 1% common error variance, .3 represents 9% common error variance and .5 represents 25% common error variance across both measurements. Given that the absolute magnitude of the variances did not affect TRC behavior (cf. Study 1), the analysis was restricted to variance ratios corresponding to reliability values ranging from 0.60 to 0.95 in increments of 0.05. Sample sizes and repetitions per condition were the same as in Study 1. Again, mean and standard deviation were reported across all 1000 conditions. Thus, the manipulated variables in Study 2 were as follows:

(1) Sample size: 2 to 1000
(2) True score stability: 1, .9, .8, .7, and .6
(3) Error score dependence: Correlations of .1, .3, and .5 between *ε*_1_ and *ε*_2_
(4) Variance ratio (true score to error score): 0.6 to 0.95 in steps of .05

### Study 2: Results

**Figure 3.**
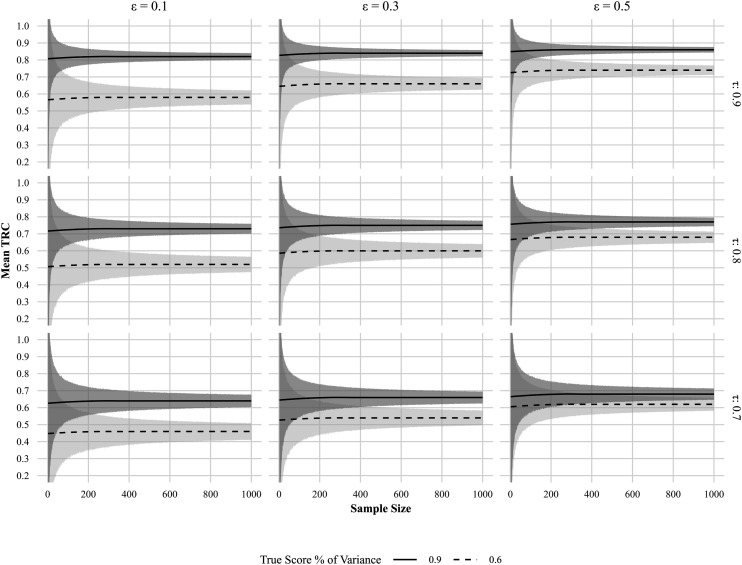
Mean estimated correlations (mean TRC) across increasing sample sizes for two levels of reliability (0.9, 0.8, and 0.7). Shaded ribbons represent 95% confidence intervals. Panels depict varying levels of true score stability (τ) and error dependency (ε).

Figure 3 illustrates how the TRC evolves as a function of τ -stability and error dependence by comparing an instrument with *“excellent”* reliability to one with *“unacceptable”* reliability. Two qualitative trends emerge. First, across all conditions, the presence of dependent error terms systematically biases the TRC upwards. This inflation is more pronounced for the low-reliability instrument, causing the TRC estimates for the two instruments to converge. As a result, differences in measurement quality become increasingly difficult to detect. Second, the homogenizing effect of error dependence intensifies as τ -stability decreases. As shown in Study 1, reductions in τ -stability tend to shift the TRC downward. However, when error dependence is introduced, this downward trend is offset, and the inflationary effect becomes more substantial under low τ -stability conditions.

**Figure 4.**
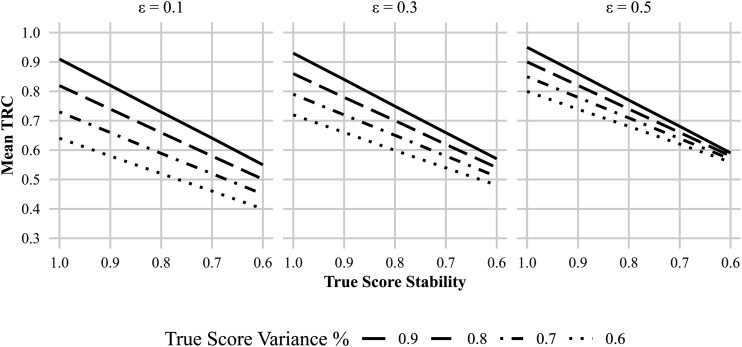
Mean estimated correlations (mean TRC) at a fixed sample size of 1000, plotted across levels of true score stability. Lines represent different levels of reliability (0.6–0.9), with more dotted lines indicating lower reliability. Panels vary by error dependency (ε).

These two trends are further quantified in Figure 4, which summarizes the average distortions in TRC estimates across varying levels of τ -stability and error dependence. As can be seen at a τ -stability of .7 and an error of .5, the different levels of reliability are impossible to tell apart.

## Discussion

The present study investigated the performance of the TRC under different conditions of variance ratios, sample sizes, true score stability, and error score dependences. Two simulations were conducted. The first study assessed how the TRC performs across different sample sizes, variance ratios, and levels of true score stability. The second study explored the effects of sample size, variance ratio, τ -stability, and error dependence on the TRC.

### Average Estimation and Bias of the TRC

Multiple findings stand out in relation to the average estimation of the TRC. When the assumptions of fixed true scores and independent errors are met, the TRC is an almost perfect estimator of the ratio of true score to error score variance, even at small sample sizes (cf. Table 1).

When true score stability decreases, the TRC estimation deviates from the variance ratio as predicted mathematically in equation (10). The expected TRC equals the product of trait stability and measurement reliability. The practical consequence is that highly reliable instruments may appear to perform poorly when the underlying trait shows only moderate stability. For instance, as shown in Table 2, a measurement with a true reliability of .9 and τ-stability of .7 yields a TRC of just .63—a downward bias of .27 relative to its true value. Though the nominal degree of bias is lower for measurements with lower reliability, the point of true score stability at which a *“good”* or even *“acceptable”* measurement becomes nominally *“unacceptable”* is reached faster. Regardless, as seen in Figure 2, by τ -stability = .8 all measurements except the measurement with excellent reliability would be deemed unacceptable. These findings demonstrate that the TRC is extremely unrobust to violations of τ -stability, as even a reduction by .2 can shift an otherwise good measurement into the *“unacceptable”* category, thus rendering conventional TRC guidelines unsuitable whenever true scores are less than perfectly stable.

When error scores are not independent, interpreting the TRC becomes significantly more difficult. Two key trends emerge under error dependence. First, TRC estimates across different levels of measurement quality begin to converge, reducing the discriminability between instruments with varying degrees of reliability. Second, this homogenizing effect becomes more pronounced as true score stability decreases. Both patterns are consistent with the theoretical predictions developed in equation (14), where the expected TRC is modeled as a weighted sum of true score stability and systematic error, each scaled by its contribution to the total variance.

These patterns introduce two core interpretive problems. First, a measurement with low reliability can appear deceptively strong if enough systematic error is present. For example, as shown in Figure 4, a poor instrument can yield a TRC similar to that of a highly reliable one if τ-stability is low (e.g., τ = .6) and systematic error is moderate to high (e.g., ε = .5). Second, the homogenizing effect itself undermines the usefulness of the TRC as a comparative index. Once systematic error is introduced, the TRC values collapse toward a narrow range, thereby eroding their ability to meaningfully distinguish between strong and weak measures.

This poses a fundamental challenge. Without separate estimates of τ-stability and systematic error, low TRC values are uninterpretable as they may reflect low reliability, low stability, or both. Conversely, a high TRC may not reflect strong measurement properties, but merely the compensatory influence of systematic error. This ambiguity is especially severe at lower levels of trait stability, where systematic error exerts disproportionate influence, and thus further distorts the reliability estimate. In such cases, the TRC loses its interpretive value entirely.

### Coefficient Stability

As seen in Table 2, the TRC stabilizes remarkably fast when true scores are perfectly stable and error is zero. Under these ideal conditions, fewer than 20 participants are required to achieve good coefficient stability ( $SD_{TRC}$ < 0.05), provided that measurement reliability is excellent. However, achieving high precision ( $SD_{TRC}$ < 0.025) under the same conditions demands nearly 200 participants. As τ -stability declines, required sample sizes increase sharply. For example, at τ -stability = .60, even measurements with *“excellent”* reliability (variance ratio = .90) require over 700 participants to reach excellent coefficient stability, with many combinations exceeding the 1,000-participant ceiling imposed by the simulation.

These sample size demands stand in stark contrast to both standard recommendations and common research practice. For instance, De Vet et al. (2011) propose 50 participants as a general benchmark, while many studies report test–retest reliability using samples of 30–100. The current results indicate that such sizes are only sufficient when trait stability exceeds .90, reliability is good (≥.80), and precision requirements are modest. Although this suggests that small sample sizes may suffice under ideal conditions, such conditions cannot be known in advance. As ideal conditions cannot be known in advance, sample sizes must plan for less favorable scenarios. The present results therefore demonstrate that much larger samples are generally required to ensure that reliability estimates are accurate and robust across a range of plausible measurement conditions.

Contrary to intuition, the TRC stabilizes faster when systematic error is introduced. However, this observed stability can be misleading as it occurs under conditions where the measurement is inherently less reliable. Systematic error furthermore increases the homogeneity of distributions, regardless of τ -stability. This trend parallels the effects observed in the mean estimation. Systematic error causes the overlap between distributions to increase, further diminishing the ability to distinguish between variance ratios. Due to this homogenization effect, it is clear that the perfect amount of systematic error should always be zero, as even under stable conditions an error of *ε* = .5 immensely reduces the confidence in the estimation.

### Feasibility

As discussed above, if the assumptions underpinning the TRC are violated, the coefficient no longer refers to reliability in any meaningful psychometric sense. The issue, however, extends beyond bias. It becomes a question of feasibility. Even in principle, can reliability be estimated when true scores are unstable or when systematic error is present?

To address this, it is important to distinguish between bias and identifiability. Bias refers to the degree an estimator diverges from the true parameter it seeks to estimate (Lehmann & Casella, 1998). Identifiability, by contrast, concerns whether the estimator produces unique results that can be clearly attributed to the true parameter. An estimator is identifiable when the number of observed (known) variables is equal to or exceeds the number of unknowns (Lewbel, 2019). With only two observed scores, it is impossible to isolate more than two latent components (Coleman, 1968; Heise, 1969; Rogosa, 2013). The TRC framework implicitly involves four latent components: measurement reliability, occasion-specific error, latent-trait stability, and systematic measurement error. Of these, the latter two are assumed to be one and zero, respectively, thereby rendering the TRC mathematically identifiable under its standard assumptions.

However, when the assumptions of score stability and error independence are not met, a principal problem of underidentification emerges as the number of unknowns exceeds the available information. This issue is illustrated in Figure 2. A measurement with excellent reliability becomes nominally unreliable at a stability level of approximately .65. The same TRC (∼.60) could also result from a measure with acceptable reliability and a stability of ∼ .75 or, as in Study 2, from a measure with poor reliability, a stability of ∼ .80, and a systematic error of .3. Because the researcher lacks direct access to these latent components and is attempting to estimate all of them simultaneously, it is impossible to identify which combination of measurement reliability, occasion-specific error, latent-trait stability, and systematic measurement error produced the observed TRC. In other words, because the TRC relies on both perfectly stable true scores and error independence, violating either assumption does not merely introduce bias, a systematic distortion, but fundamentally renders the model mathematically unidentifiable. The more assumptions that are violated, the more severely the TRC becomes underidentified.

This limitation would be less problematic if the underlying assumptions held reliably. However, as discussed in section 3, no psychological trait exhibits perfect temporal stability, and many fall below the threshold ( r < .80) at which instability substantially distorts TRC estimates. The way the TRC is applied in practice also exacerbates these issues. Often tests are validated using undergraduate samples, with participants completing the same instrument at the beginning and again at the end of a semester. Incentives such as course credit are often used to recruit participation (e.g., Quirin et al., 2009; Spreng et al., 2009).

While convenient, this approach is riddled with confounding influences. Testing environments are typically uncontrolled, increasing the likelihood of systematic error through contextual variation or motivational shifts. Moreover, the psychological state of students changes considerably over the course of a semester. Midterms, deadlines, fatigue, and fluctuating personal circumstances all contribute to transient changes in affect, cognition, and behavior, the very domains in which psychological traits are typically measured. These sources of variation are not just noise. They represent systematic, temporally structured influences on measurement. Ironically, the further apart the two testing occasions are spaced, a common practice to reduce memory effects, the more likely it becomes that the latent variable itself has changed (Polit, 2014). This creates a methodological impasse. Testing too close risks recall bias, and testing too far apart risks violating stability. The TRC is thus caught in a catch-22, where its assumptions can only be satisfied under conditions that undermine the study design itself.

Furthermore, stability cannot be verified using the same dataset from which reliability is estimated. With only two time points, observed changes remain ambiguous, potentially reflecting random error, systematic bias, or genuine shifts in the latent trait. The direction and magnitude of this change are likewise unknowable. This epistemic limitation is absolute. No internal diagnostic within the coefficient can reveal which source predominates. The same, of course, applies to systematic error. Changes in testing conditions or psychological context cannot be detected or corrected with only two observations. If such error is present, it is absorbed silently into the observed scores, leaving no empirical trace. As a result, researchers must assume both stability and error independence despite having no empirical basis for doing so. Given the above, both assumptions can be rarely justifiable in applied contexts.

In applied psychometric work, these limitations demand caution. The TRC, when used in isolation, is insufficient as a claim about reliability. In practice, it must be contextualized by information such as internal consistency metrics, prior knowledge about trait stability, or design choices that plausibly minimize confounding variance. Even then, the estimate rests on untestable assumptions. Predictions about trait stability from longitudinal studies may offer one approach to addressing these limitations by forecasting potential biases and adjusting guidelines, but such approaches are speculative and offer no guarantee of validity in the current application.

Furthermore, as discussed earlier, the required sample size for accurate TRC estimation increases rapidly as trait stability decreases, such that *“excellent”* precision becomes infeasible at anything below τ = .80.

This makes the TRC unusable in most applied settings. Even without systematic error, adequate coefficient stability at moderate τ-stability requires hundreds of participants. For example, at τ -stability = .60, a sample size exceeding 700 is required to estimate the TRC with high precision, even when reliability is *“excellent*.” These requirements far exceed the sample sizes typically available in psychological research. Of course, alternative approaches exist that do not depend on assumptions about temporal stability, most notably internal consistency metrics such as Cronbach’s α, McDonald’s ω, or GLB. However, these statistics are not conceptually equivalent to the TRC, as they assess the common variance among items within a single test administration and are therefore orthogonal to the stability of true scores (Guttman, 1945; Zinbarg et al., 2005). More importantly, modern interpretations of these indices no longer treat them as measures of reliability, but rather as estimates of the proportion of variance attributable to a common latent factor (Revelle & Zinbarg, 2008). Thus, these statistics do not provide an alternative to the TRC.

The only viable path forward for test–retest contexts lies in abandoning the two-time-point framework and adopting models that can empirically estimate, rather than assume, key components like trait stability and systematic error. Designs incorporating three or more measurement occasions, such as the longitudinal framework proposed by Heise (1969), allow for model identification under realistic conditions. Similarly, latent variable approaches like longitudinal structural equation modeling offer a principled way to separate true change from error, though they require more complex designs and greater methodological rigor. Without such designs, or a shift toward single-administration reliability coefficients, the TRC provides not a meaningful estimate of reliability, but a mathematically ambiguous quantity that gives the illusion of psychometric certainty where none exists.

## Limitations

Several limitations of the present study should be noted. The present simulations used normally distributed continuous data analyzed with Pearson’s r , as is standard in test–retest research. This setup represents the best-case scenario. Pearson’s r performs optimally with normal data but is known to be less accurate under skewed or non-normal distributions (Bishara & Hittner, 2014; Kowalski, 1972). Thus, the nominal results for bias, stability and required sample size may differ and increase with the degree of non-normality. Nominal results may also differ when other coefficients are used (e.g., Spearman for ranks and tetrachoric for dichotomous outcomes).

Likewise, ordinal or binomial data can yield different estimates. However, in psychological measurement, summed scores from multiple items tend to approximate normal distributions by the CLT (Norman, 2010), and ordinal variables with several categories can often be treated as continuous without meaningful distortion (Rhemtulla et al., 2012).

Lastly, in line with classical test theory, the simulations assumed independence between true scores and error scores. Situations in which τ and ε covary, for example, when error is partly determined by trait level (e.g., ceiling or floor effects), were not modeled. Such covariances would introduce additional sources of systematic error and further reduce the identifiability of the TRC. Again, the results therefore represent a relatively favorable case and violations of this assumption would only strengthen the conclusion that the TRC is unidentifiable. Thus, while absolute values may vary across data types, the central conclusion remains robust. The TRC is identifiable only under an idealized scenario that is unattainable in practice.

## Conclusion

The TRC is a fundamental reliability index in CTT. It provides a straightforward method for quantifying measurement reliability by comparing two measurement points, making it a widely used tool in psychometrics. The current findings indicate that the TRC offers a robust and accurate estimate of reliability if and only if the true scores are perfectly stable ( τ ≥ .9), and no systematic error is present ( ε < .1). However, when even one of these conditions is violated, the TRC rapidly loses both interpretability and utility. Instability in true scores or the presence of systematic error not only biases the estimate but renders it fundamentally unidentifiable.

This is not merely a practical shortcoming but a structural one. With only two time points, it is mathematically impossible to separate true reliability from temporal instability and systematic error. As such, TRC-based reliability collapses under common empirical conditions, even when parameters are well specified or large samples are used. Given these limitations, the TRC should be applied only when its assumptions can be justified and when sample sizes are sufficient to enable precise estimation. In most, if not all, applied contexts, these criteria are not met.

While other reliability indices such as Cronbach’s α, McDonald’s ω, or the GLB exist, they do not resolve the limitations outlined above, as they assess internal consistency within a single test administration rather than temporal reliability. For longitudinal assessments, designs incorporating three or more time points, as proposed by Heise (1969), provide a more principled approach to estimating reliability, although they demand a level of methodological rigor not commonly observed in practice. Without such designs, the TRC does not provide a meaningful estimate of reliability but rather a false sense of certainty.

## Supplemental Material

Supplemental material - On the Unreliability of Test–Retest Reliability Supplemental material for On the Unreliability of Test–Retest Reliability by Domenic Groh in Applied Psychological Measurement.

## Data Availability

The R code used to simulate the data, as well as the results reported here are available at the Open Science Framework at https://osf.io/x7ptw.
