# Supplementary material for: On the Unreliability of Test–Retest Reliability
Source: Appl Psychol Meas. 2025 Nov 26:01466216251401213. Online ahead of print. doi: 10.1177/01466216251401213 (PMC12657207; doi:10.1177/01466216251401213)
Supplement: Supplemental material - On the Unreliability of Test–Retest Reliability [file sj-pdf-1-apm-10.1177_01466216251401213.pdf]

Supplement to “On the Unreliability of Test-Retest Reliability”

This supplement presents additional plots and tables corresponding to the simulation studies reported in the main text of “On the Unreliability of Test-Retest Reliability.” The organization of the supplement mirrors that of the main paper. Supplementary Figure 1, along with Tables 1, 2, and 3, provides further results from Simulation Study 1. Supplementary Figure 2.x, Figure 3, and Tables 4.x and 5.x present additional findings from Simulation Study 2. Each figure and table is accompanied by a brief explanatory text to aid interpretation.

Contents

Supplementary Figure 1 **2**

Supplementary Table 1 and 2 **4**

Supplementary Table 3 **7**

Supplementary Figure 2.x **8**

Supplementary Figure 3 **12**

Supplementary Table 4.x **13**

Supplementary Table 5.x **18**

Supplementary Figure 1: Explanation

This figure shows the mean correlation of the TRC, computed from 1,000 simulated samples, across a range of sample sizes (10–1,000), four reliability levels ($r_{\tau x} = 0.6$–0.9), and six levels of true-score stability ($\tau = 1.0$ to 0.5). In each panel, the solid black line is the LOESS-smoothed mean TRC, the grey ribbon its 95% confidence interval, and the dotted line (in all but the $\tau = 1$ panels) the $\tau = 1$ reference curve for direct comparison.

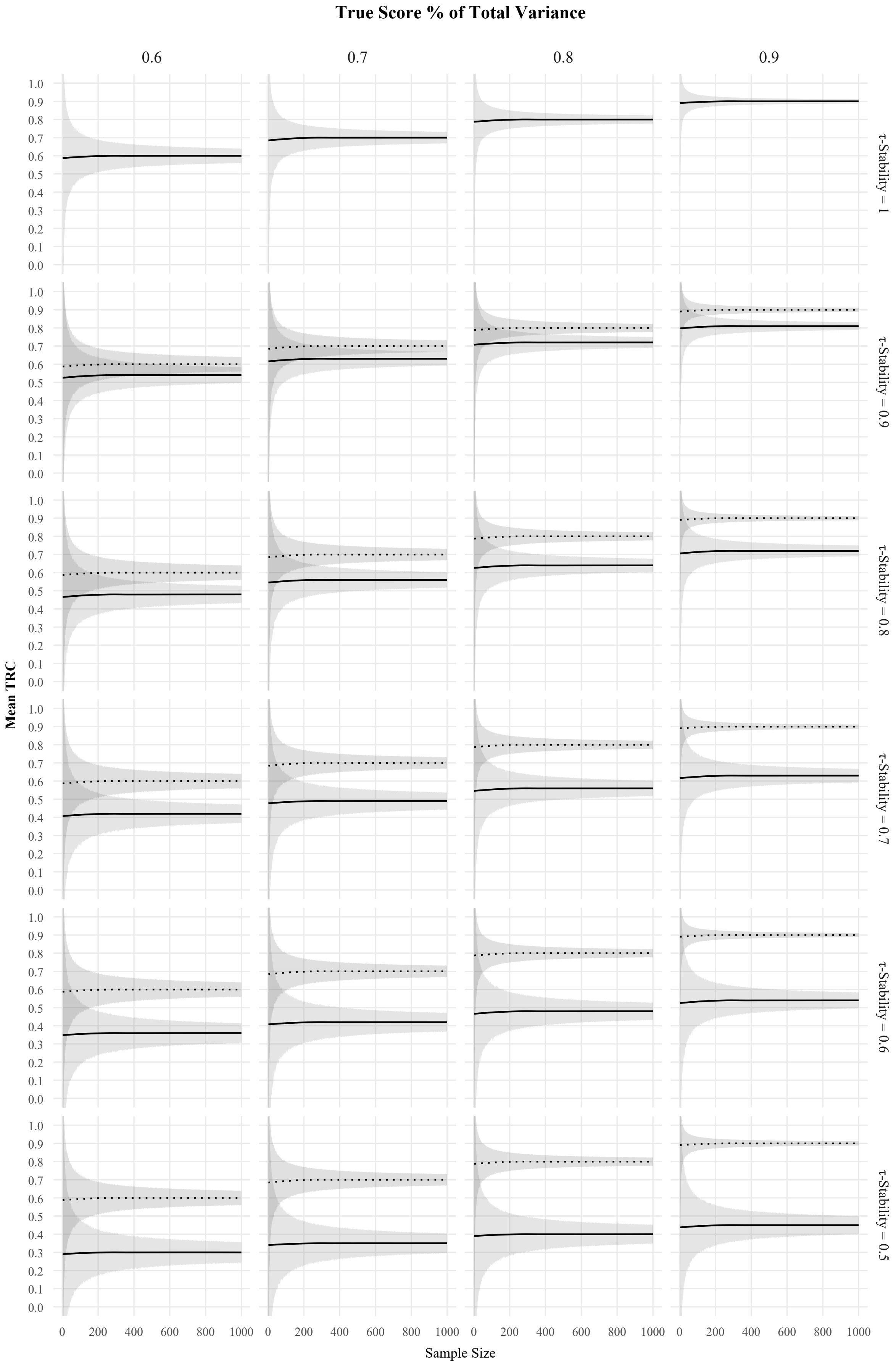

Supplementary Figure 1: Mean TRC across sample size, variance ratio, and true-score stability.

Supplementary Table 1 and 2: Explanation

The tables show how the TRC behaves as a function of stability and sample size for selected values of sample size, reliability, and stability. Table 1 shows the mean estimated TRC across all 1000 iterations. Table 2 shows the standard deviation of said mean estimations. The points where good stability ($SD < 0.05$) is reached are marked in italic; points where excellent stability ($SD < 0.025$) are marked in bold.

Supplementary Table 1. Mean correlation of the TRC across 1000 samples sorted by sample size, variance ratio and true score stability

A	n	$r_{\tau x}$ 0.6	0.7	0.8	0.9
	10	0.58	0.67	0.78	0.89
	15	0.59	0.69	0.78	0.89
	30	0.59	0.69	0.80	0.90
	50	0.60	0.70	0.80	0.90
	75	0.60	0.70	0.80	0.90
	100	0.60	0.70	0.80	0.90
	250	0.60	0.70	0.80	0.90
	500	0.60	0.70	0.80	0.90
	750	0.60	0.70	0.80	0.90
	1000	0.60	0.70	0.80	0.90

B	n	$r_{\tau x}$ 0.6	0.7	0.8	0.9
	10	0.53	0.60	0.71	0.79
	15	0.52	0.62	0.71	0.80
	30	0.53	0.62	0.71	0.81
	50	0.53	0.63	0.72	0.81
	75	0.54	0.63	0.72	0.81
	100	0.54	0.63	0.72	0.81
	250	0.54	0.63	0.72	0.81
	500	0.54	0.63	0.72	0.81
	750	0.54	0.63	0.72	0.81
	1000	0.54	0.63	0.72	0.81

C	n	$r_{\tau x}$ 0.6	0.7	0.8	0.9
	10	0.46	0.53	0.61	0.69
	15	0.47	0.55	0.62	0.71
	30	0.46	0.55	0.63	0.72
	50	0.47	0.55	0.63	0.72
	75	0.47	0.56	0.64	0.72
	100	0.48	0.56	0.64	0.72
	250	0.48	0.56	0.64	0.72
	500	0.48	0.56	0.64	0.72
	750	0.48	0.56	0.64	0.72
	1000	0.48	0.56	0.64	0.72

D	n	$r_{\tau x}$ 0.6	0.7	0.8	0.9
	10	0.41	0.46	0.53	0.62
	15	0.42	0.48	0.55	0.61
	30	0.41	0.49	0.56	0.62
	50	0.42	0.48	0.56	0.63
	75	0.41	0.49	0.56	0.63
	100	0.42	0.49	0.56	0.62
	250	0.42	0.49	0.56	0.63
	500	0.42	0.49	0.56	0.63
	750	0.42	0.49	0.56	0.63
	1000	0.42	0.49	0.56	0.63

E	n	$r_{\tau x}$ 0.6	0.7	0.8	0.9
	10	0.34	0.41	0.45	0.51
	15	0.34	0.40	0.47	0.54
	30	0.36	0.41	0.48	0.53
	50	0.36	0.42	0.48	0.53
	75	0.35	0.42	0.48	0.54
	100	0.36	0.42	0.48	0.54
	250	0.36	0.42	0.48	0.54
	500	0.36	0.42	0.48	0.54
	750	0.36	0.42	0.48	0.54
	1000	0.36	0.42	0.48	0.54

F	n	$r_{\tau x}$ 0.6	0.7	0.8	0.9
	10	0.28	0.33	0.38	0.44
	15	0.29	0.34	0.38	0.43
	30	0.31	0.35	0.39	0.44
	50	0.30	0.35	0.40	0.44
	75	0.30	0.35	0.40	0.44
	100	0.30	0.35	0.40	0.44
	250	0.30	0.35	0.40	0.45
	500	0.30	0.35	0.40	0.45
	750	0.30	0.35	0.40	0.45
	1000	0.30	0.35	0.40	0.45

n: Sample size
$r_{\tau x}$: Square root of true reliability
A: Stable true score, **B**: 0.9 stability, **C**: 0.8 stability, **D**: 0.7 stability, **E**: 0.6 stability, **F**: 0.5 stability

Supplementary Table 2. Standard deviation of the TRC across 1000 Samples sorted by sample size, variance ratio and true score stability

**A**	n	$r_{\tau x}$	0.6	0.7	0.8	0.9
	10		0.23	0.19	0.15	0.08
	15		0.19	0.14	0.11	0.06
	30		0.12	0.10	0.07	*0.04*
	50		0.10	0.07	0.05	0.03
	75		0.07	0.06	*0.04*	**0.02**
	100		0.06	0.05	0.04	0.02
	250		*0.04*	*0.03*	**0.02**	0.01
	500		0.03	**0.02**	0.02	0.01
	750		**0.02**	0.02	0.01	0.01
	1000		0.02	0.02	0.01	0.01

**B**	n	$r_{\tau x}$	0.6	0.7	0.8	0.9
	10		0.25	0.23	0.17	0.15
	15		0.20	0.17	0.14	0.10
	30		0.14	0.12	0.09	0.07
	50		0.10	0.09	0.07	0.05
	75		0.08	0.07	0.06	*0.04*
	100		0.07	0.06	0.05	0.03
	250		0.05	*0.04*	*0.03*	**0.02**
	500		*0.03*	0.03	**0.02**	0.02
	750		0.03	**0.02**	0.02	0.01
	1000		**0.02**	0.02	0.02	0.01

**C**	n	$r_{\tau x}$	0.6	0.7	0.8	0.9
	10		0.27	0.25	0.22	0.19
	15		0.21	0.20	0.17	0.13
	30		0.15	0.14	0.12	0.09
	50		0.12	0.10	0.09	0.07
	75		0.09	0.08	0.07	0.06
	100		0.08	0.07	0.06	0.05
	250		0.05	*0.04*	*0.04*	*0.03*
	500		*0.04*	0.03	0.03	**0.02**
	750		0.03	**0.02**	**0.02**	0.02
	1000		**0.02**	0.02	0.02	0.02

**D**	n	$r_{\tau x}$	0.6	0.7	0.8	0.9
	10		0.29	0.28	0.25	0.21
	15		0.21	0.22	0.19	0.17
	30		0.16	0.14	0.13	0.12
	50		0.12	0.11	0.10	0.09
	75		0.10	0.09	0.08	0.07
	100		0.09	0.08	0.07	0.06
	250		0.05	0.05	*0.04*	*0.04*
	500		*0.04*	*0.03*	0.03	0.03
	750		0.03	0.03	0.03	**0.02**
	1000		0.03	**0.02**	**0.02**	0.02

**E**	n	$r_{\tau x}$	0.6	0.7	0.8	0.9
	10		0.30	0.28	0.27	0.25
	15		0.24	0.23	0.21	0.20
	30		0.16	0.15	0.14	0.13
	50		0.13	0.12	0.11	0.10
	75		0.10	0.10	0.09	0.09
	100		0.09	0.09	0.08	0.07
	250		0.05	0.05	0.05	*0.04*
	500		*0.04*	*0.04*	*0.03*	0.03
	750		0.03	0.03	0.03	0.03
	1000		0.03	0.03	**0.02**	**0.02**

**F**	n	$r_{\tau x}$	0.6	0.7	0.8	0.9
	10		0.31	0.30	0.29	0.28
	15		0.24	0.24	0.23	0.22
	30		0.17	0.16	0.16	0.15
	50		0.13	0.13	0.12	0.12
	75		0.11	0.10	0.10	0.09
	100		0.09	0.09	0.08	0.08
	250		0.06	0.06	0.05	0.05
	500		*0.04*	*0.04*	*0.04*	*0.04*
	750		0.03	0.03	0.03	0.03
	1000		0.03	0.03	0.03	**0.02**

n: Sample size
$r_{\tau x}$: Square root of true reliability
A: Stable true score, **B**: 0.9 stability, **C**: 0.8 stability, **D**: 0.7 stability, **E**: 0.6 stability, **F**: 0.5 stability
Points where good stability is reached are marked in italic; points where excellent stability is reached are marked in bold

Supplementary Table 3: Explanation

The table below compares distinct variance-ratio pairs that yield equivalent theoretical reliability values, evaluating whether differences in absolute ratio magnitude influence the resulting TRC estimates across sample sizes of 10, 15, 30, and 50.

Supplementary Table 3. Comparison of distinct variance-ratio pairs that yield equivalent theoretical reliability values, across four sample sizes

**Variance Ratio**		1:1	9:9	3:2	9:6	2:1	8:4	3:1	9:3	4:1	8:2
Sample Size											
10											
	Mean r	0.48	0.47	0.58	0.56	0.65	0.64	0.73	0.73	0.79	0.78
	Mean SD	0.27	0.27	0.23	0.25	0.21	0.22	0.18	0.17	0.15	0.15
15											
	Mean r	0.48	0.49	0.58	0.58	0.65	0.66	0.74	0.73	0.79	0.78
	Mean SD	0.22	0.21	0.18	0.18	0.16	0.16	0.13	0.13	0.11	0.11
30											
	Mean r	0.50	0.49	0.60	0.59	0.67	0.66	0.75	0.75	0.80	0.80
	Mean SD	0.15	0.14	0.12	0.12	0.10	0.11	0.08	0.08	0.07	0.07
50											
	Mean r	0.49	0.50	0.59	0.60	0.66	0.66	0.75	0.74	0.80	0.80
	Mean SD	0.11	0.11	0.09	0.09	0.08	0.08	0.06	0.07	0.05	0.05
100											
	Mean r	0.50	0.50	0.60	0.60	0.67	0.66	0.75	0.75	0.80	0.80
	Mean SD	0.08	0.07	0.07	0.06	0.05	0.06	0.05	0.05	0.04	0.04

Supplementary Figure 2.x: Explanation

The figure shows the mean correlation of the TRC, computed from 1,000 simulated samples, across a range of sample sizes (2–1,000), four levels of error dependence ($\varepsilon = 0$, 0.1, 0.3 and 0.5), and five levels of true-score stability ($\tau = 1.0$ to 0.5). The figure is split into three parts. In each part a measurement of "excellent" reliability ($r_{\tau x} = 0.9$) is contrasted with successively worse measurements. In Supplementary Figure 2.1 the comparison is made with a "good" measurement ($r_{\tau x} = 0.8$), in 2.2 with an "acceptable" measurement ($r_{\tau x} = 0.7$) and in 2.3 with an "unacceptable" measurement ($r_{\tau x} = 0.6$). In each panel, the solid black line is the LOESS smoothed mean TRC of the "exellent" measurement, the gray ribbon its 95% confidence interval, while the dotted line represents the comparison of the respective "good", "acceptable" and "unacceptable" measurements.

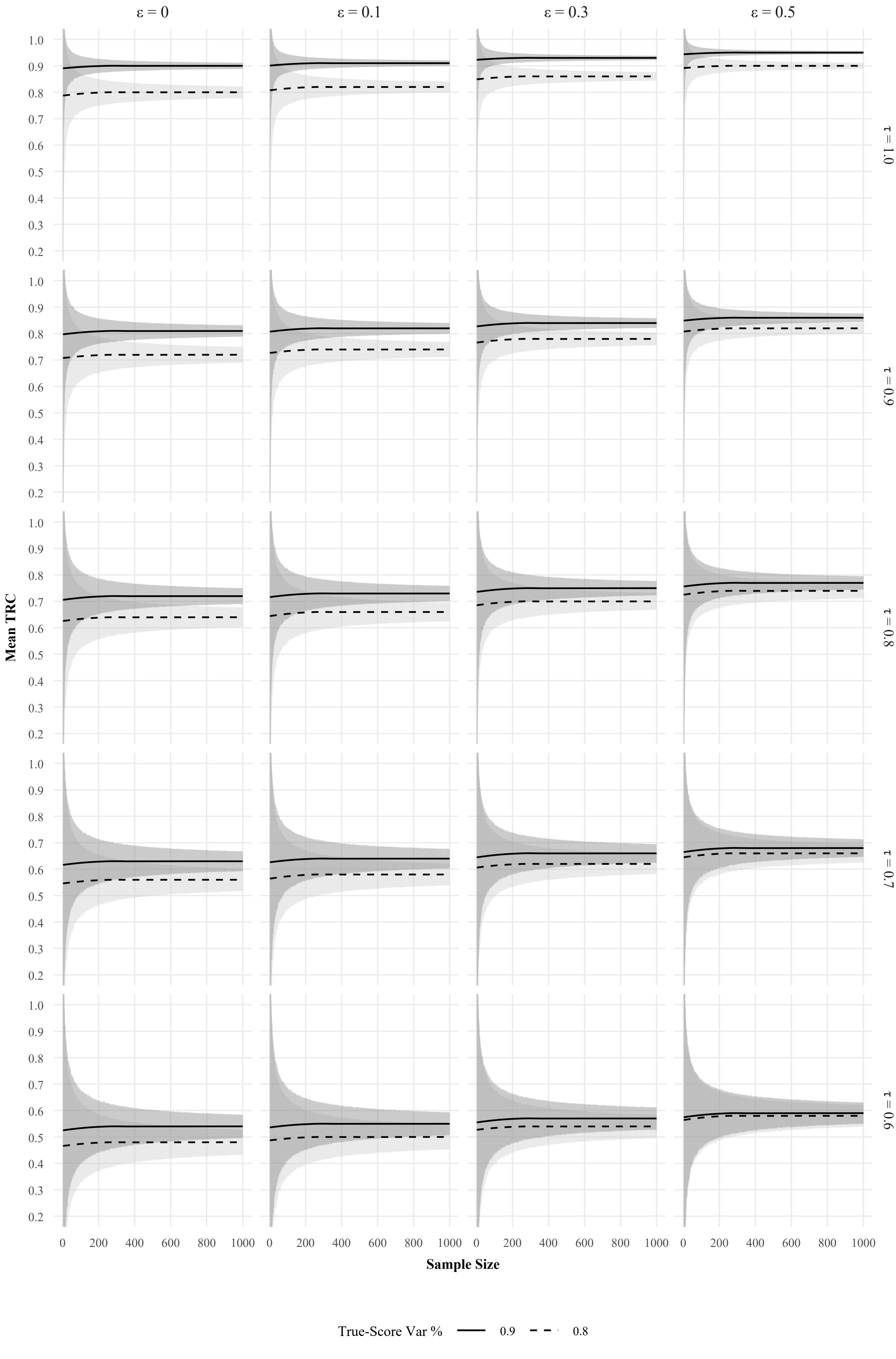

Supplementary Figure 2.1: Mean TRC across sample size, variance ratio, true-score stability and error dependence for a "good" and "excellent" measurement.

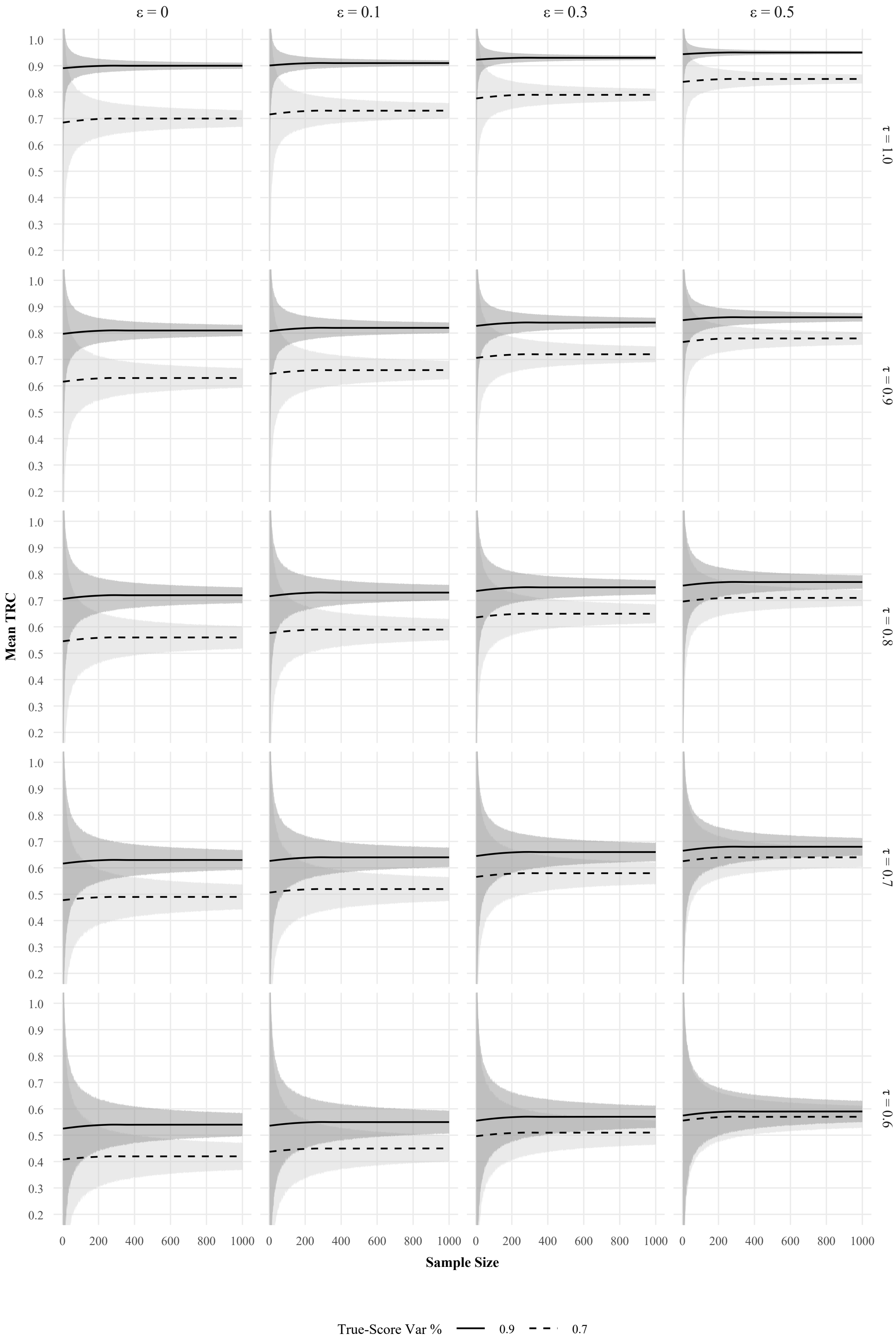

Supplementary Figure 2.2: Mean TRC across sample size, variance ratio, true-score stability and error dependence for an "acceptable" and "excellent" measurement.

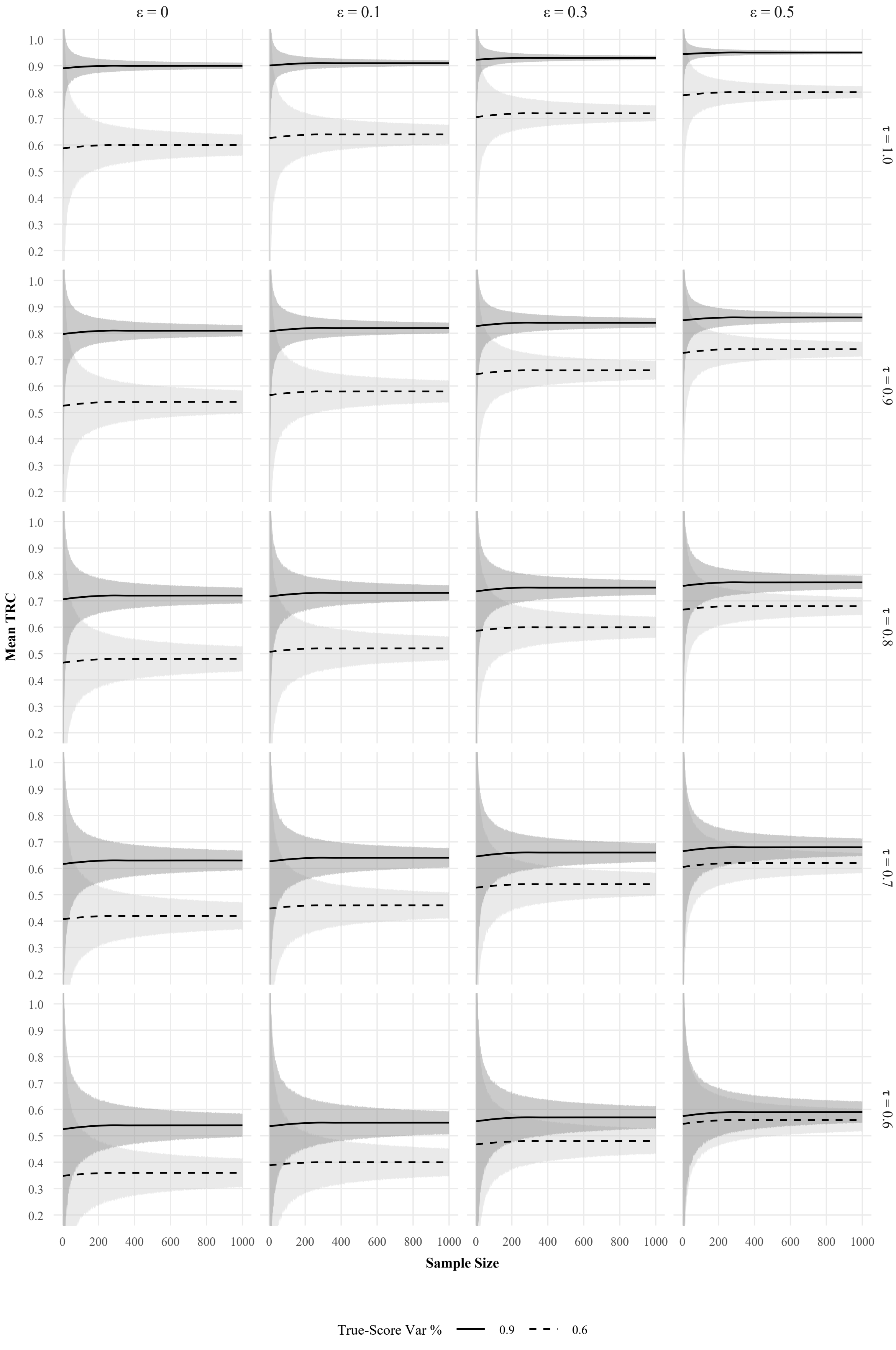

Supplementary Figure 2.3: Mean TRC across sample size, variance ratio, true-score stability and error dependence for an “unacceptable” and “excellent” measurement.

Supplementary Figure 3: Explanation

This figure shows a direct combination of Figure 2 from Study 1 and Figure 4 from Study 2. Since Study 1 used independent errors, it is assigned as $\varepsilon = 0$.

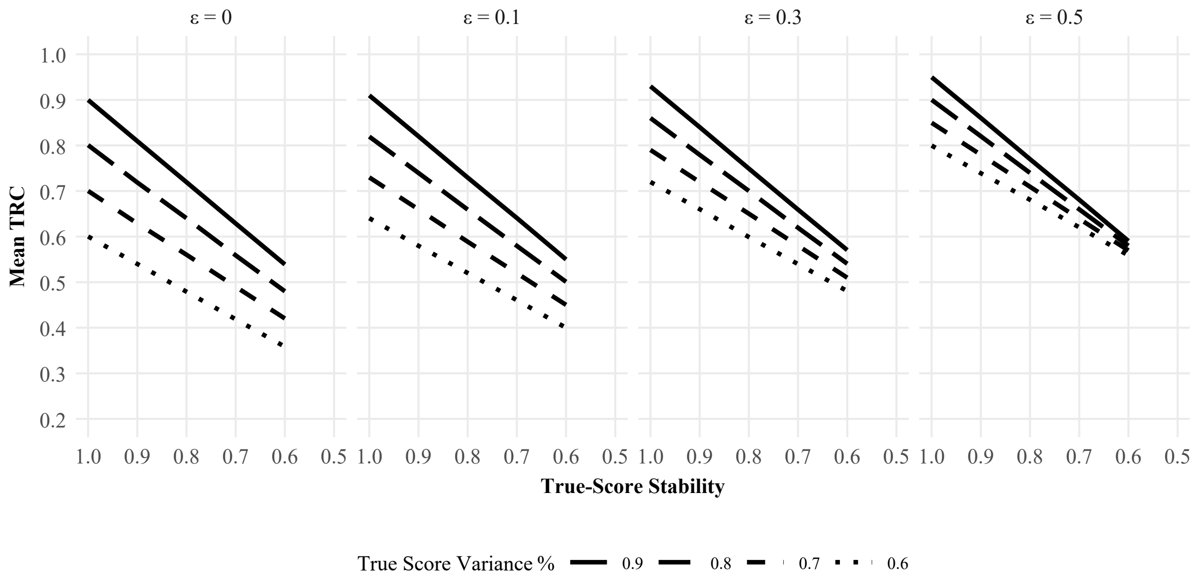

Supplementary Figure 3: Mean estimated correlations (Mean TRC) at a fixed sample size of 1000, plotted across levels of true-score stability. Lines represent different levels of reliability (0.6–0.9), with more dotted lines indicating lower reliability. Panels vary by error dependency (ε).

Supplementary Table 4.x and 5.x: Explanation

The tables show how the TRC behaves as a function of stability and sample size for selected values of sample size, reliability, and stability. Table 1 shows the mean estimated TRC across all 1000 iterations. Table 2 shows the standard deviation of said mean estimations. The points where good stability (SD < 0.05) is reached are marked in *italic*; points where excellent stability (SD < 0.025) are marked in **bold**.

Supplementary Table 4.1. Mean of the TRC across 1000 Samples sorted by sample size, variance ratio, true score stability and systematic error (**τ: 1 ε: .1** to **τ: .9 ε: .1**)

**τ: 1 ε: .1**	n	$r_{\tau x}$	0.6	0.65	0.70	0.75	0.8	0.85	0.90	0.95
	10		0.61	0.65	0.70	0.77	0.81	0.85	0.90	0.95
	15		0.62	0.67	0.72	0.76	0.81	0.86	0.91	0.95
	30		0.64	0.69	0.73	0.77	0.81	0.86	0.91	0.95
	50		0.63	0.68	0.73	0.77	0.82	0.86	0.91	0.95
	75		0.64	0.68	0.73	0.77	0.82	0.86	0.91	0.95
	100		0.64	0.68	0.73	0.77	0.82	0.86	0.91	0.95
	250		0.64	0.68	0.73	0.77	0.82	0.86	0.91	0.95
	500		0.64	0.68	0.73	0.77	0.82	0.86	0.91	0.95
	750		0.64	0.69	0.73	0.77	0.82	0.87	0.91	0.96
	1000		0.64	0.68	0.73	0.78	0.82	0.87	0.91	0.95
**τ: 1 ε: .3**	n	$r_{\tau x}$	0.6	0.65	0.70	0.75	0.8	0.85	0.90	0.95
	10		0.70	0.73	0.77	0.80	0.85	0.88	0.92	0.96
	15		0.71	0.73	0.78	0.81	0.85	0.89	0.92	0.96
	30		0.71	0.75	0.78	0.82	0.86	0.89	0.93	0.96
	50		0.71	0.75	0.79	0.82	0.86	0.90	0.93	0.96
	75		0.72	0.75	0.79	0.83	0.86	0.89	0.93	0.96
	100		0.72	0.76	0.79	0.83	0.86	0.89	0.93	0.96
	250		0.72	0.75	0.79	0.82	0.86	0.89	0.93	0.96
	500		0.72	0.76	0.79	0.82	0.86	0.89	0.93	0.96
	750		0.72	0.75	0.79	0.82	0.86	0.89	0.93	0.96
	1000		0.72	0.76	0.79	0.82	0.86	0.89	0.93	0.97
**τ: 1 ε: .5**	n	$r_{\tau x}$	0.6	0.65	0.70	0.75	0.8	0.85	0.90	0.95
	10		0.78	0.80	0.83	0.87	0.89	0.92	0.94	0.97
	15		0.79	0.81	0.84	0.87	0.89	0.92	0.95	0.97
	30		0.80	0.82	0.84	0.87	0.90	0.92	0.95	0.97
	50		0.80	0.82	0.85	0.87	0.90	0.92	0.95	0.98
	75		0.80	0.82	0.85	0.87	0.90	0.92	0.95	0.97
	100		0.80	0.82	0.85	0.87	0.90	0.92	0.95	0.97
	250		0.80	0.82	0.85	0.87	0.90	0.92	0.95	0.97
	500		0.80	0.82	0.85	0.87	0.90	0.92	0.95	0.98
	750		0.80	0.83	0.85	0.88	0.90	0.92	0.95	0.98
	1000		0.80	0.82	0.85	0.87	0.90	0.93	0.95	0.98
**τ: .9 ε: .1**	n	$r_{\tau x}$	0.6	0.65	0.70	0.75	0.8	0.85	0.90	0.95
	10		0.54	0.61	0.63	0.67	0.72	0.76	0.80	0.85
	15		0.57	0.61	0.65	0.68	0.73	0.77	0.81	0.86
	30		0.57	0.61	0.66	0.70	0.74	0.77	0.81	0.86
	50		0.58	0.61	0.66	0.69	0.74	0.77	0.82	0.86
	75		0.58	0.62	0.66	0.70	0.74	0.78	0.82	0.86
	100		0.58	0.62	0.66	0.70	0.74	0.78	0.82	0.86
	250		0.58	0.62	0.66	0.70	0.74	0.78	0.82	0.86
	500		0.58	0.62	0.66	0.70	0.74	0.78	0.82	0.86
	750		0.58	0.62	0.66	0.70	0.74	0.78	0.82	0.86
	1000		0.58	0.62	0.66	0.70	0.74	0.78	0.82	0.86

n: Sample size
$r_{\tau x}$: Square root of true reliability
τ: Level of true score stability, ε: Level of error score correlation

Supplementary Table 4.2. Mean of the TRC across 1000 Samples sorted by sample size, variance ratio, true score stability and systematic error (**τ: .9 ε: .3** to **τ: .8 ε: .3**)

**τ: .9 ε: .3**	n	$r_{\tau x}$	0.6	0.65	0.70	0.75	0.8	0.85	0.90	0.95
	10		0.65	0.67	0.71	0.73	0.76	0.79	0.82	0.86
	15		0.65	0.67	0.71	0.74	0.77	0.80	0.83	0.86
	30		0.65	0.69	0.72	0.74	0.77	0.80	0.84	0.86
	50		0.65	0.69	0.71	0.75	0.78	0.81	0.84	0.87
	75		0.66	0.69	0.72	0.75	0.78	0.81	0.84	0.87
	100		0.66	0.69	0.72	0.75	0.78	0.81	0.84	0.87
	250		0.66	0.69	0.72	0.75	0.78	0.81	0.84	0.87
	500		0.66	0.69	0.72	0.75	0.78	0.81	0.84	0.87
	750		0.66	0.69	0.72	0.75	0.78	0.81	0.84	0.87
	1000		0.66	0.69	0.72	0.75	0.78	0.81	0.84	0.87

**τ: .9 ε: .5**	n	$r_{\tau x}$	0.6	0.65	0.70	0.75	0.8	0.85	0.90	0.95
	10		0.72	0.73	0.76	0.78	0.79	0.82	0.85	0.87
	15		0.73	0.75	0.77	0.79	0.81	0.83	0.85	0.87
	30		0.73	0.75	0.77	0.79	0.81	0.83	0.86	0.88
	50		0.74	0.76	0.78	0.80	0.82	0.84	0.86	0.88
	75		0.74	0.76	0.78	0.80	0.82	0.84	0.86	0.88
	100		0.74	0.76	0.78	0.80	0.82	0.84	0.86	0.88
	250		0.74	0.76	0.78	0.80	0.82	0.84	0.86	0.88
	500		0.74	0.76	0.78	0.80	0.82	0.84	0.86	0.88
	750		0.74	0.76	0.78	0.80	0.82	0.84	0.86	0.88
	1000		0.74	0.76	0.78	0.80	0.82	0.84	0.86	0.88

**τ: .8 ε: .1**	n	$r_{\tau x}$	0.6	0.65	0.70	0.75	0.8	0.85	0.90	0.95
	10		0.50	0.54	0.56	0.61	0.64	0.67	0.71	0.74
	15		0.52	0.54	0.58	0.62	0.64	0.67	0.72	0.75
	30		0.51	0.55	0.59	0.62	0.66	0.69	0.72	0.77
	50		0.51	0.55	0.59	0.63	0.66	0.69	0.73	0.76
	75		0.52	0.55	0.59	0.63	0.65	0.69	0.73	0.76
	100		0.52	0.55	0.59	0.62	0.66	0.69	0.73	0.76
	250		0.52	0.56	0.59	0.63	0.66	0.69	0.73	0.76
	500		0.52	0.56	0.59	0.62	0.66	0.69	0.73	0.76
	750		0.52	0.55	0.59	0.62	0.66	0.69	0.73	0.76
	1000		0.52	0.55	0.59	0.62	0.66	0.70	0.73	0.76

**τ: .8 ε: .3**	n	$r_{\tau x}$	0.6	0.65	0.70	0.75	0.8	0.85	0.90	0.95
	10		0.59	0.60	0.64	0.66	0.68	0.71	0.73	0.76
	15		0.59	0.61	0.63	0.65	0.69	0.71	0.74	0.77
	30		0.59	0.62	0.64	0.67	0.70	0.72	0.75	0.78
	50		0.59	0.62	0.65	0.67	0.70	0.72	0.75	0.77
	75		0.60	0.62	0.65	0.67	0.69	0.72	0.75	0.77
	100		0.60	0.62	0.65	0.67	0.70	0.72	0.75	0.77
	250		0.60	0.63	0.65	0.67	0.70	0.72	0.75	0.77
	500		0.60	0.62	0.65	0.67	0.70	0.73	0.75	0.77
	750		0.60	0.63	0.65	0.68	0.70	0.73	0.75	0.77
	1000		0.60	0.63	0.65	0.68	0.70	0.72	0.75	0.77

n: Sample size
$r_{\tau x}$: Square root of true reliability
τ: Level of true score stability, ε: Level of error score correlation

Supplementary Table 4.3. Mean of the TRC across 1000 Samples sorted by sample size, variance ratio, true score stability and systematic error (**τ: .8 ε: .5** to **τ: .7 ε: .5**)

**τ: .8 ε: .5**	n	$r_{\tau x}$	0.6	0.65	0.70	0.75	0.8	0.85	0.90	0.95
	10		0.65	0.67	0.68	0.71	0.71	0.73	0.74	0.76
	15		0.66	0.68	0.69	0.71	0.72	0.73	0.75	0.77
	30		0.68	0.69	0.71	0.71	0.73	0.76	0.76	0.78
	50		0.68	0.69	0.71	0.72	0.74	0.75	0.77	0.78
	75		0.68	0.69	0.70	0.72	0.74	0.75	0.77	0.78
	100		0.68	0.69	0.71	0.72	0.74	0.75	0.77	0.78
	250		0.68	0.69	0.71	0.73	0.74	0.75	0.77	0.79
	500		0.68	0.70	0.71	0.72	0.74	0.75	0.77	0.79
	750		0.68	0.70	0.71	0.72	0.74	0.75	0.77	0.79
	1000		0.68	0.69	0.71	0.72	0.74	0.75	0.77	0.79
**τ: .7 ε: .1**	n	$r_{\tau x}$	0.6	0.65	0.70	0.75	0.8	0.85	0.90	0.95
	10		0.43	0.48	0.49	0.52	0.55	0.58	0.61	0.65
	15		0.44	0.47	0.50	0.53	0.57	0.60	0.62	0.66
	30		0.45	0.49	0.51	0.54	0.57	0.61	0.63	0.66
	50		0.45	0.49	0.52	0.54	0.57	0.60	0.64	0.66
	75		0.46	0.49	0.52	0.54	0.58	0.61	0.64	0.67
	100		0.46	0.49	0.52	0.55	0.58	0.61	0.64	0.67
	250		0.46	0.49	0.52	0.55	0.58	0.61	0.64	0.67
	500		0.46	0.49	0.52	0.55	0.58	0.61	0.64	0.67
	750		0.46	0.49	0.52	0.55	0.58	0.61	0.64	0.67
	1000		0.46	0.49	0.52	0.55	0.58	0.61	0.64	0.67
**τ: .7 ε: .3**	n	$r_{\tau x}$	0.6	0.65	0.70	0.75	0.8	0.85	0.90	0.95
	10		0.52	0.53	0.57	0.58	0.59	0.62	0.63	0.67
	15		0.52	0.54	0.57	0.59	0.61	0.63	0.64	0.66
	30		0.53	0.55	0.57	0.59	0.61	0.63	0.65	0.68
	50		0.53	0.55	0.58	0.59	0.62	0.64	0.66	0.67
	75		0.54	0.56	0.57	0.60	0.62	0.63	0.66	0.68
	100		0.53	0.56	0.58	0.60	0.62	0.64	0.66	0.68
	250		0.54	0.56	0.58	0.60	0.62	0.64	0.66	0.68
	500		0.54	0.56	0.58	0.60	0.62	0.64	0.66	0.68
	750		0.54	0.56	0.58	0.60	0.62	0.64	0.66	0.68
	1000		0.54	0.56	0.58	0.60	0.62	0.64	0.66	0.68
**τ: .7 ε: .5**	n	$r_{\tau x}$	0.6	0.65	0.70	0.75	0.8	0.85	0.90	0.95
	10		0.61	0.61	0.61	0.63	0.64	0.65	0.66	0.67
	15		0.61	0.61	0.62	0.63	0.64	0.65	0.67	0.68
	30		0.61	0.63	0.64	0.65	0.65	0.66	0.67	0.68
	50		0.61	0.63	0.64	0.65	0.65	0.67	0.68	0.69
	75		0.61	0.63	0.64	0.65	0.66	0.67	0.68	0.69
	100		0.62	0.63	0.64	0.65	0.66	0.67	0.68	0.69
	250		0.62	0.63	0.64	0.65	0.66	0.67	0.68	0.69
	500		0.62	0.63	0.64	0.65	0.66	0.67	0.68	0.69
	750		0.62	0.63	0.64	0.65	0.66	0.67	0.68	0.69
	1000		0.62	0.63	0.64	0.65	0.66	0.67	0.68	0.69

n: Sample size
$r_{\tau x}$: Square root of true reliability
τ: Level of true score stability, ε: Level of error score correlation

Supplementary Table 4.4. Mean of the TRC across 1000 Samples sorted by sample size, variance ratio, true score stability and systematic error (**τ: .6 ε: .1** to **τ: .6 ε: .5**)

**τ: .6 ε: .1**	n	$r_{\tau x}$	0.6	0.65	0.70	0.75	0.8	0.85	0.90	0.95
	10		0.37	0.41	0.42	0.46	0.48	0.50	0.52	0.56
	15		0.40	0.41	0.44	0.45	0.50	0.51	0.54	0.57
	30		0.39	0.42	0.45	0.47	0.51	0.52	0.54	0.57
	50		0.40	0.42	0.45	0.47	0.50	0.53	0.54	0.56
	75		0.40	0.42	0.45	0.47	0.50	0.52	0.55	0.57
	100		0.40	0.42	0.45	0.48	0.50	0.52	0.55	0.58
	250		0.40	0.42	0.45	0.47	0.50	0.53	0.55	0.57
	500		0.40	0.43	0.45	0.47	0.50	0.53	0.55	0.57
	750		0.40	0.43	0.45	0.48	0.50	0.53	0.55	0.57
	1000		0.40	0.42	0.45	0.47	0.50	0.52	0.55	0.57

**τ: .6 ε: .3**	n	$r_{\tau x}$	0.6	0.65	0.70	0.75	0.8	0.85	0.90	0.95
	10		0.47	0.48	0.51	0.50	0.53	0.52	0.55	0.57
	15		0.47	0.47	0.50	0.52	0.52	0.54	0.56	0.57
	30		0.48	0.48	0.51	0.52	0.53	0.55	0.56	0.57
	50		0.47	0.49	0.50	0.52	0.54	0.55	0.57	0.58
	75		0.47	0.50	0.50	0.52	0.54	0.55	0.57	0.58
	100		0.48	0.49	0.51	0.52	0.54	0.55	0.57	0.58
	250		0.48	0.49	0.51	0.53	0.54	0.55	0.57	0.59
	500		0.48	0.50	0.51	0.53	0.54	0.56	0.57	0.59
	750		0.48	0.49	0.51	0.52	0.54	0.55	0.57	0.58
	1000		0.48	0.50	0.51	0.52	0.54	0.55	0.57	0.58

**τ: .6 ε: .5**	n	$r_{\tau x}$	0.6	0.65	0.70	0.75	0.8	0.85	0.90	0.95
	10		0.54	0.53	0.55	0.55	0.55	0.56	0.56	0.57
	15		0.55	0.54	0.56	0.57	0.57	0.56	0.57	0.58
	30		0.55	0.55	0.56	0.57	0.57	0.57	0.58	0.59
	50		0.56	0.56	0.57	0.57	0.57	0.58	0.58	0.59
	75		0.56	0.56	0.56	0.57	0.58	0.59	0.59	0.60
	100		0.56	0.56	0.57	0.57	0.58	0.58	0.59	0.60
	250		0.56	0.57	0.57	0.57	0.58	0.58	0.59	0.59
	500		0.56	0.56	0.57	0.57	0.58	0.58	0.59	0.59
	750		0.56	0.56	0.57	0.58	0.58	0.59	0.59	0.60
	1000		0.56	0.56	0.57	0.57	0.58	0.59	0.59	0.60

n: Sample size
$r_{\tau x}$: Square root of true reliability
τ: Level of true score stability, ε: Level of error score correlation

Supplementary Table 5.1. Standard deviation of the TRC across 1000 Samples sorted by sample size, variance ratio, true score stability and systematic error (**τ: 1 ε: .1** to **τ: .9 ε: .1**)

**τ: 1 ε: .1**	n	$r_{\tau x}$	0.6	0.65	0.70	0.75	0.8	0.85	0.90	0.95
	10		0.23	0.21	0.19	0.15	0.13	0.11	0.07	*0.04*
	15		0.17	0.16	0.14	0.12	0.10	0.09	0.06	0.03
	30		0.11	0.10	0.09	0.08	0.07	0.05	*0.03*	**0.02**
	50		0.08	0.08	0.07	0.06	0.05	*0.04*	0.03	0.01
	75		0.07	0.06	0.06	0.05	*0.04*	0.03	**0.02**	0.01
	100		0.06	0.06	*0.05*	*0.04*	0.03	0.03	0.02	0.01
	250		*0.04*	*0.03*	0.03	0.03	**0.02**	**0.02**	0.01	0.01
	500		0.03	**0.02**	**0.02**	**0.02**	0.01	0.01	0.01	0.00
	750		**0.02**	0.02	0.02	0.01	0.01	0.01	0.01	0.00
	1000		0.02	0.02	0.01	0.01	0.01	0.01	0.01	0.00

**τ: 1 ε: .3**	n	$r_{\tau x}$	0.6	0.65	0.70	0.75	0.8	0.85	0.90	0.95
	10		0.18	0.17	0.15	0.14	0.11	0.08	0.06	0.03
	15		0.15	0.13	0.11	0.10	0.09	0.06	0.05	**0.02**
	30		0.10	0.08	0.07	0.06	0.05	*0.04*	*0.03*	0.01
	50		0.07	0.06	0.06	0.05	*0.04*	0.03	**0.02**	0.01
	75		0.06	0.05	*0.04*	*0.04*	0.03	**0.02**	0.02	0.01
	100		0.05	*0.04*	0.04	0.03	0.03	0.02	0.01	0.01
	250		*0.03*	0.03	**0.02**	**0.02**	**0.02**	0.01	0.01	0.00
	500		**0.02**	**0.02**	0.02	0.01	0.01	0.01	0.01	0.00
	750		0.02	0.02	0.01	0.01	0.01	0.01	0.00	0.00
	1000		0.01	0.01	0.01	0.01	0.01	0.01	0.00	0.00

**τ: 1 ε: .5**	n	$r_{\tau x}$	0.6	0.65	0.70	0.75	0.8	0.85	0.90	0.95
	10		0.14	0.14	0.11	0.09	0.09	0.06	0.05	**0.02**
	15		0.11	0.10	0.08	0.07	0.06	*0.04*	*0.03*	0.02
	30		0.07	0.06	0.05	0.05	*0.04*	0.03	**0.02**	0.01
	50		0.06	0.05	*0.04*	*0.04*	0.03	**0.02**	0.01	0.01
	75		*0.04*	*0.04*	0.03	0.03	**0.02**	0.02	0.01	0.01
	100		0.04	0.03	0.03	**0.02**	0.02	0.01	0.01	0.01
	250		**0.02**	**0.02**	**0.02**	0.01	0.01	0.01	0.01	0.00
	500		0.02	0.01	0.01	0.01	0.01	0.01	0.00	0.00
	750		0.01	0.01	0.01	0.01	0.01	0.01	0.00	0.00
	1000		0.01	0.01	0.01	0.01	0.01	0.00	0.00	0.00

**τ: .9 ε: .1**	n	$r_{\tau x}$	0.6	0.65	0.70	0.75	0.8	0.85	0.90	0.95
	10		0.26	0.23	0.22	0.20	0.17	0.17	0.14	0.10
	15		0.18	0.18	0.16	0.15	0.13	0.12	0.10	0.08
	30		0.12	0.12	0.11	0.09	0.09	0.08	0.07	0.05
	50		0.10	0.09	0.08	0.08	0.07	0.06	0.05	*0.04*
	75		0.08	0.07	0.07	0.06	0.05	0.05	*0.04*	0.03
	100		0.07	0.06	0.06	0.05	0.05	*0.04*	0.03	0.03
	250		*0.04*	*0.04*	*0.03*	*0.03*	*0.03*	**0.02**	**0.02**	**0.02**
	500		0.03	0.03	0.03	**0.02**	**0.02**	0.02	0.01	0.01
	750		**0.02**	**0.02**	**0.02**	0.02	0.02	0.01	0.01	0.01
	1000		0.02	0.02	0.02	0.02	0.01	0.01	0.01	0.01

n: Sample size
$r_{\tau x}$: Square root of true reliability
τ: Level of true score stability, ε: Level of error score correlation
Points where good stability is reached are marked in italic; points where excellent stability is reached are marked in bold

Supplementary Table 5.2. Standard deviation of the TRC across 1000 Samples sorted by sample size, variance ratio, true score stability and systematic error (**τ: .9 ε: .3** to **τ: .8 ε: .3**)

**τ: .9 ε: .3**	n	$r_{\tau x}$	0.6	0.65	0.70	0.75	0.8	0.85	0.90	0.95
	10		0.21	0.20	0.18	0.18	0.16	0.14	0.13	0.10
	15		0.16	0.16	0.14	0.13	0.12	0.10	0.09	0.08
	30		0.11	0.11	0.09	0.09	0.08	0.07	0.06	0.05
	50		0.08	0.07	0.07	0.06	0.06	0.05	*0.04*	*0.04*
	75		0.07	0.06	0.06	0.05	0.05	*0.04*	0.03	0.03
	100		0.05	0.05	0.05	*0.04*	*0.04*	0.04	0.03	**0.02**
	250		*0.04*	*0.03*	*0.03*	0.03	0.03	**0.02**	**0.02**	0.02
	500		**0.02**	**0.02**	**0.02**	**0.02**	**0.02**	0.02	0.01	0.01
	750		0.02	0.02	0.02	0.02	0.01	0.01	0.01	0.01
	1000		0.02	0.02	0.01	0.01	0.01	0.01	0.01	0.01

**τ: .9 ε: .5**	n	$r_{\tau x}$	0.6	0.65	0.70	0.75	0.8	0.85	0.90	0.95
	10		0.19	0.18	0.16	0.15	0.14	0.13	0.11	0.09
	15		0.13	0.12	0.12	0.11	0.10	0.09	0.08	0.07
	30		0.09	0.08	0.08	0.07	0.06	0.06	0.05	0.05
	50		0.07	0.06	0.06	0.05	0.05	*0.04*	*0.04*	*0.03*
	75		0.05	0.05	0.05	*0.04*	*0.04*	0.04	0.03	0.03
	100		0.05	*0.04*	*0.04*	0.04	0.03	0.03	0.03	**0.02**
	250		*0.03*	0.03	0.03	**0.02**	**0.02**	**0.02**	**0.02**	0.01
	500		**0.02**	**0.02**	**0.02**	0.02	0.01	0.01	0.01	0.01
	750		0.02	0.02	0.01	0.01	0.01	0.01	0.01	0.01
	1000		0.01	0.01	0.01	0.01	0.01	0.01	0.01	0.01

**τ: .8 ε: .1**	n	$r_{\tau x}$	0.6	0.65	0.70	0.75	0.8	0.85	0.90	0.95
	10		0.26	0.25	0.23	0.22	0.21	0.20	0.18	0.16
	15		0.19	0.20	0.19	0.17	0.17	0.16	0.13	0.12
	30		0.14	0.14	0.12	0.12	0.11	0.10	0.09	0.08
	50		0.11	0.10	0.10	0.09	0.08	0.08	0.07	0.06
	75		0.09	0.08	0.08	0.07	0.07	0.06	0.06	0.05
	100		0.07	0.07	0.07	0.06	0.06	0.05	0.05	*0.04*
	250		0.05	*0.04*	*0.04*	*0.04*	*0.04*	*0.03*	*0.03*	0.03
	500		*0.03*	0.03	0.03	0.03	0.03	**0.02**	**0.02**	**0.02**
	750		0.03	**0.02**	**0.02**	**0.02**	**0.02**	0.02	0.02	0.02
	1000		**0.02**	0.02	0.02	0.02	0.02	0.02	0.01	0.01

**τ: .8 ε: .3**	n	$r_{\tau x}$	0.6	0.65	0.70	0.75	0.8	0.85	0.90	0.95
	10		0.23	0.24	0.20	0.21	0.20	0.18	0.17	0.15
	15		0.18	0.18	0.17	0.16	0.14	0.14	0.13	0.12
	30		0.12	0.12	0.11	0.11	0.10	0.10	0.08	0.08
	50		0.10	0.09	0.09	0.08	0.07	0.07	0.07	0.06
	75		0.08	0.07	0.07	0.06	0.06	0.05	0.05	0.05
	100		0.06	0.06	0.06	0.05	0.05	0.05	*0.04*	*0.04*
	250		*0.04*	*0.04*	*0.04*	*0.04*	*0.03*	*0.03*	0.03	**0.02**
	500		0.03	0.03	0.03	**0.02**	**0.02**	**0.02**	**0.02**	0.02
	750		**0.02**	**0.02**	**0.02**	0.02	0.02	0.02	0.02	0.01
	1000		0.02	0.02	0.02	0.02	0.02	0.02	0.01	0.01

n: Sample size
$r_{\tau x}$: Square root of true reliability
τ: Level of true score stability, ε: Level of error score correlation
Points where good stability is reached are marked in italic; points where excellent stability is reached are marked in bold

Supplementary Table 5.3. Standard deviation of the TRC across 1000 Samples sorted by sample size, variance ratio, true score stability and systematic error (**τ: .8 ε: .5** to **τ: .7 ε: .5**)

**τ: .8 ε: .5**	n	$r_{\tau x}$	0.6	0.65	0.70	0.75	0.8	0.85	0.90	0.95
	10		0.20	0.20	0.20	0.18	0.18	0.17	0.17	0.14
	15		0.15	0.15	0.15	0.14	0.14	0.13	0.12	0.11
	30		0.10	0.10	0.09	0.09	0.09	0.08	0.08	0.08
	50		0.08	0.08	0.07	0.07	0.07	0.06	0.06	0.06
	75		0.06	0.06	0.06	0.05	0.06	0.05	0.05	0.05
	100		0.05	0.05	0.05	0.05	0.05	*0.04*	*0.04*	*0.04*
	250		*0.04*	*0.03*	*0.03*	*0.03*	*0.03*	0.03	0.03	**0.02**
	500		**0.02**	**0.02**	**0.02**	**0.02**	**0.02**	**0.02**	**0.02**	0.02
	750		0.02	0.02	0.02	0.02	0.02	0.02	0.01	0.01
	1000		0.02	0.02	0.02	0.02	0.01	0.01	0.01	0.01

**τ: .7 ε: .1**	n	$r_{\tau x}$	0.6	0.65	0.70	0.75	0.8	0.85	0.90	0.95
	10		0.29	0.27	0.27	0.26	0.25	0.24	0.22	0.21
	15		0.22	0.21	0.21	0.20	0.19	0.17	0.18	0.15
	30		0.14	0.14	0.14	0.13	0.13	0.12	0.11	0.11
	50		0.12	0.11	0.11	0.10	0.09	0.10	0.09	0.08
	75		0.09	0.09	0.08	0.08	0.08	0.07	0.07	0.07
	100		0.08	0.08	0.07	0.07	0.07	0.06	0.06	0.06
	250		0.05	0.05	0.05	0.05	*0.04*	*0.04*	*0.04*	*0.04*
	500		*0.03*	*0.03*	*0.03*	*0.03*	0.03	0.03	0.03	**0.02**
	750		0.03	0.03	0.03	0.03	**0.02**	**0.02**	**0.02**	0.02
	1000		0.03	0.02	**0.02**	**0.02**	0.02	0.02	0.02	0.02

**τ: .7 ε: .3**	n	$r_{\tau x}$	0.6	0.65	0.70	0.75	0.8	0.85	0.90	0.95
	10		0.26	0.25	0.24	0.23	0.24	0.22	0.21	0.20
	15		0.19	0.19	0.18	0.18	0.17	0.17	0.16	0.16
	30		0.14	0.13	0.12	0.13	0.12	0.11	0.11	0.10
	50		0.10	0.10	0.09	0.10	0.09	0.09	0.08	0.08
	75		0.08	0.08	0.08	0.07	0.07	0.07	0.07	0.07
	100		0.07	0.07	0.07	0.07	0.06	0.06	0.06	0.06
	250		*0.04*	*0.04*	*0.04*	*0.04*	*0.04*	*0.04*	*0.04*	*0.04*
	500		0.03	0.03	0.03	0.03	0.03	0.03	0.03	**0.02**
	750		0.03	**0.02**	**0.02**	**0.02**	**0.02**	**0.02**	**0.02**	0.02
	1000		**0.02**	0.02	0.02	0.02	0.02	0.02	0.02	0.02

**τ: .7 ε: .5**	n	$r_{\tau x}$	0.6	0.65	0.70	0.75	0.8	0.85	0.90	0.95
	10		0.21	0.21	0.21	0.21	0.21	0.20	0.21	0.19
	15		0.17	0.18	0.16	0.17	0.17	0.16	0.15	0.16
	30		0.12	0.12	0.11	0.10	0.11	0.11	0.10	0.10
	50		0.09	0.09	0.09	0.09	0.08	0.08	0.08	0.08
	75		0.07	0.07	0.07	0.07	0.07	0.06	0.06	0.06
	100		0.06	0.06	0.06	0.06	0.06	0.06	0.05	0.05
	250		*0.04*	*0.04*	*0.04*	*0.04*	*0.04*	*0.04*	*0.03*	*0.03*
	500		0.03	0.03	0.03	0.03	0.03	0.03	**0.02**	**0.02**
	750		**0.02**	**0.02**	**0.02**	**0.02**	**0.02**	**0.02**	0.02	0.02
	1000		0.02	0.02	0.02	0.02	0.02	0.02	0.02	0.02

n: Sample size
$r_{\tau x}$: Square root of true reliability
τ: Level of true score stability, ε: Level of error score correlation
Points where good stability is reached are marked in italic; points where excellent stability is reached are marked in bold

Supplementary Table 5.4. Standard deviation of the TRC across 1000 Samples sorted by sample size, variance ratio, true score stability and systematic error (**τ: .6 ε: .1** to **τ: .6 ε: .5**)

**τ: .6 ε: .1**	n	$r_{\tau x}$	0.6	0.65	0.70	0.75	0.8	0.85	0.90	0.95
	10		0.30	0.29	0.28	0.28	0.27	0.26	0.25	0.24
	15		0.23	0.22	0.22	0.21	0.21	0.20	0.20	0.19
	30		0.15	0.15	0.14	0.14	0.14	0.14	0.13	0.13
	50		0.12	0.12	0.11	0.12	0.11	0.10	0.10	0.09
	75		0.10	0.10	0.10	0.09	0.09	0.09	0.08	0.08
	100		0.09	0.08	0.08	0.08	0.08	0.08	0.07	0.07
	250		0.05	0.05	0.05	0.05	0.05	*0.04*	*0.04*	*0.04*
	500		*0.04*	*0.04*	*0.04*	*0.04*	*0.03*	0.03	0.03	0.03
	750		0.03	0.03	0.03	0.03	0.03	0.03	0.03	**0.02**
	1000		0.03	0.03	0.03	0.03	**0.02**	**0.02**	**0.02**	0.02
**τ: .6 ε: .3**	n	$r_{\tau x}$	0.6	0.65	0.70	0.75	0.8	0.85	0.90	0.95
	10		0.27	0.26	0.25	0.27	0.25	0.26	0.25	0.23
	15		0.21	0.21	0.21	0.20	0.21	0.19	0.20	0.19
	30		0.14	0.16	0.13	0.14	0.14	0.13	0.12	0.12
	50		0.11	0.11	0.11	0.10	0.10	0.10	0.10	0.09
	75		0.09	0.09	0.09	0.08	0.08	0.08	0.08	0.08
	100		0.08	0.08	0.08	0.07	0.07	0.07	0.07	0.07
	250		0.05	0.05	0.05	0.05	0.05	*0.04*	*0.04*	*0.04*
	500		*0.03*	*0.03*	*0.03*	*0.03*	*0.03*	0.03	0.03	0.03
	750		0.03	0.03	0.03	0.03	0.03	0.03	**0.02**	**0.02**
	1000		**0.02**	**0.02**	**0.02**	**0.02**	**0.02**	**0.02**	0.02	0.02
**τ: .6 ε: .5**	n	$r_{\tau x}$	0.6	0.65	0.70	0.75	0.8	0.85	0.90	0.95
	10		0.27	0.26	0.25	0.25	0.25	0.24	0.25	0.23
	15		0.19	0.20	0.19	0.19	0.20	0.19	0.19	0.18
	30		0.14	0.13	0.13	0.12	0.13	0.13	0.12	0.13
	50		0.10	0.10	0.10	0.10	0.10	0.09	0.10	0.10
	75		0.08	0.08	0.08	0.08	0.08	0.08	0.08	0.08
	100		0.07	0.07	0.07	0.07	0.07	0.07	0.07	0.06
	250		*0.04*	*0.04*	*0.04*	*0.04*	*0.04*	*0.04*	*0.04*	*0.04*
	500		0.03	0.03	0.03	0.03	0.03	0.03	0.03	0.03
	750		0.02	0.03	0.03	**0.02**	**0.02**	**0.02**	**0.02**	**0.02**
	1000		**0.02**	**0.02**	**0.02**	0.02	0.02	0.02	0.02	0.02

n: Sample size
$r_{\tau x}$: Square root of true reliability
τ: Level of true score stability, ε: Level of error score correlation
Points where good stability is reached are marked in italic; points where excellent stability is reached are marked in bold
